# Predicting cognitive scores from wearable-based digital physiological features using machine learning: data from a clinical trial in mild cognitive impairment

**DOI:** 10.1186/s12916-024-03252-y

**Published:** 2024-01-25

**Authors:** Yuri G. Rykov, Michael D. Patterson, Bikram A. Gangwar, Syaheed B. Jabar, Jacklyn Leonardo, Kok Pin Ng, Nagaendran Kandiah

**Affiliations:** 1 Neuroglee Therapeutics, Singapore, Singapore; 2 Department of Neurology, National Neuroscience Institute, Singapore, Singapore; 3 Duke-NUS Medical School, Singapore, Singapore; 4 Dementia Research Centre, Lee Kong Chian School of Medicine, Nanyang Technological University, Singapore, Singapore

**Keywords:** Digital physiological features, Digital biomarkers, Wearable sensor data, Mild cognitive impairment, Remote patient monitoring, Machine learning

## Abstract

**Background:**

Continuous assessment and remote monitoring of cognitive function in individuals with mild cognitive impairment (MCI) enables tracking therapeutic effects and modifying treatment to achieve better clinical outcomes. While standardized neuropsychological tests are inconvenient for this purpose, wearable sensor technology collecting physiological and behavioral data looks promising to provide proxy measures of cognitive function. The objective of this study was to evaluate the predictive ability of digital physiological features, based on sensor data from wrist-worn wearables, in determining neuropsychological test scores in individuals with MCI.

**Methods:**

We used the dataset collected from a 10-week single-arm clinical trial in older adults (50–70 years old) diagnosed with amnestic MCI (*N* = 30) who received a digitally delivered multidomain therapeutic intervention. Cognitive performance was assessed before and after the intervention using the Neuropsychological Test Battery (NTB) from which composite scores were calculated (executive function, processing speed, immediate memory, delayed memory and global cognition). The Empatica E4, a wrist-wearable medical-grade device, was used to collect physiological data including blood volume pulse, electrodermal activity, and skin temperature. We processed sensors’ data and extracted a range of physiological features. We used interpolated NTB scores for 10-day intervals to test predictability of scores over short periods and to leverage the maximum of wearable data available. In addition, we used individually centered data which represents deviations from personal baselines. Supervised machine learning was used to train models predicting NTB scores from digital physiological features and demographics. Performance was evaluated using “leave-one-subject-out” and “leave-one-interval-out” cross-validation.

**Results:**

The final sample included 96 aggregated data intervals from 17 individuals. In total, 106 digital physiological features were extracted. We found that physiological features, especially measures of heart rate variability, correlated most strongly to the executive function compared to other cognitive composites. The model predicted the actual executive function scores with correlation *r* = 0.69 and intra-individual changes in executive function scores with *r* = 0.61.

**Conclusions:**

Our findings demonstrated that wearable-based physiological measures, primarily HRV, have potential to be used for the continuous assessments of cognitive function in individuals with MCI.

**Supplementary Information:**

The online version contains supplementary material available at 10.1186/s12916-024-03252-y.

## Background

Mild cognitive impairment (MCI) is a noticeable decline in an individual’s cognitive function beyond that expected by aging alone and remains an important issue to address since the estimated global prevalence of MCI in older adults (aged 50 and older) is 15.6% [1]. MCI is often a sign of prodromic dementia, where further cognitive decline may still be prevented or even reversed [2]. However, since MCI can be caused by multiple etiologies [3], it is not always clear which treatment will be effective. Thus, to address this problem, interventions can benefit from continuous measurement of response to treatment for key outcomes that will allow alterations and adjustments in treatment to achieve better clinical outcomes and higher cost-effectiveness by dropping ineffective components [4, 5]. However, existing neuropsychological tests developed to assess cognition and diagnose dementia and MCI, while well-validated, have several limitations that restrict their usefulness for continuous assessment and monitoring. These standardized tests are time-consuming, lack ecological validity (performed outside of the context of everyday patients’ activities), require trained staff to administer them, and likely have insufficient test–retest reliability if frequently administered (e.g. due to learning effects and lack of alternate test versions). Although digital assessments can mitigate some of these issues, individuals must allocate time to complete them [6]. An ideal alternative assessment would work passively and automatically without any effort from the individual being tested. Mobile and wearable technologies have emerged as this possible alternative in providing a solution for remote, passive, and continuous assessment of cognitive function based on behavioral and physiological data [7, 8].

The predominant biological signal acquired from wearable sensors is blood volume pulse used for measuring heart rate (HR) and pulse/heart rate variability (HRV). HRV metrics are based on the time variation between successive heartbeats and indicate the sympathovagal balance representing the equilibrium between the two branches of the autonomic nervous system (ANS), the sympathetic (fight-or-flight) and parasympathetic (rest-and-digest) [9]. Recent systematic meta-analytic reviews show an association between cognitive functioning and HRV in both the general population and those with neurodegenerative disorders [10–12] supporting the heart-brain axis [13, 14], which is part of a two-way circuit between the central and autonomic nervous systems. Previous studies have indicated that specific HRV metrics, namely the high-frequency band of the HRV power spectrum (HRV-HF), the root mean of the square successive differences (RMSSD), and respiratory sinus arrhythmia (RSA), exhibit a small yet statistically significant positive correlation (*r* = 0.25) with global cognitive functioning in individuals with neurodegenerative conditions. Resting-state HRV showed a slightly stronger effect (*r* = 0.29) [12]. While these correlations are still too small to be useful in predicting exact cognitive scores, novel physiological features may show stronger correlations which would demonstrate more utility.

Attempts to determine how HRV affects specific areas of cognition have challenged researchers because the links are multifarious and dependent on many possibly mediating factors. For example, complex cognition involves more neural activity than simple cognitive tasks and thus requires more metabolic resources such as oxygenated blood [15, 16]. HRV could be an indirect index of the ability of the heart to quickly adapt to the current metabolic needs of the brain. However, HRV was positively correlated with executive function (*r* = 0.19) in both healthy and neurodegenerative condition populations as shown in two independent meta-analyses [11, 12]. Furthermore, HRV may also link to episodic memory as evidenced by a recent study reporting that a dynamic change in HRV as response to a sympathetic challenge is associated with a decline in episodic memory few years later [17].

The existing body of evidence primarily stems from electrocardiography conducted with specialized medical equipment in clinical settings, and largely because of this, it consists of cross-sectional studies employing between-subject designs [18]. Alternatively, photoplethysmography (PPG) sensors inbuilt in wearable devices can also accurately measure time intervals between heartbeats required for HRV calculation and, at the same time, allow continuous data collection over longer periods of time enabling longitudinal designs. Nevertheless, to the best of our knowledge, no research has evaluated the association between PPG-based HRV and cognitive performance longitudinally.

Other potential digital physiological proxies of cognitive function that can be collected with wearables are electrodermal activity (EDA) and skin temperature. EDA measures skin conductance/resistance which varies with sweat secretion and may indicate physiological arousal or stress [19]. Prior research exploring differences in EDA under different controlled conditions in healthy populations found that both tonic and phasic skin conductance measures are sensitive to cognitive stressors [20, 21]. The phasic component of EDA, reflecting rapid skin conductance fluctuations, was strongly correlated to certain brainwaves related to performance in an error awareness cognitive tasks, and with the decline in performance among individuals who experienced sleep deprivation [22]. However, these effects on EDA were task-induced and were transient in nature. EDA collected passively has been under-examined for measuring cognitive abilities. Another physiological parameter is skin (or body) temperature. Recent research has revealed that lower temperatures measured during 12 h of habitual daily activities were significantly associated with improved performance on various cognitive tasks [23]. This included measures of inhibitory control and semantic verbal fluency. Furthermore, higher long-term peak-to-peak temperature amplitudes correlated with better cognitive performance in both healthy controls and individuals with MCI [23]. These findings support the hypothesis that age-associated thermoregulatory deficits and metabolic disruptions could be involved in Alzheimer’s disease (AD) pathogenesis [24].

Not only do novel digital physiological features demonstrate promise for tracking cognition, but newly developed data-driven analysis techniques and machine learning also can amplify this potential. For example, one study showed that models based on passive data from interactions with smartphones predicted scores of neuropsychological tests with correlations ranging from 0.62 to 0.83 [7]. Moreover, another study demonstrated that features based on passive smartphone usage and smartwatch sensor data improved accuracy of discriminating MCI from healthy individuals compared to models based on demographics alone [25]. Additionally, high accuracy was achieved in prognosing MCI progression to dementia by using machine learning and neuro-motor parameters collected during performance of dedicated augmented reality tasks with a smartphone/tablet [26]. Thus, machine learning (ML) methods applied to wearable data may be useful for predicting cognitive function.

In summary, the research on passive digital physiological features for measuring cognitive performance is important because it can enable early detection, provide more objective measures that are not influenced by test–retest effects, allow better remote continuous monitoring of the effects of interventions, and be more cost-effective than clinic-based assessments. The current body of evidence largely came from cross-sectional data collected using medical equipment, such as electrocardiography, in clinical settings. Therefore, there is a lack of longitudinal data collected with wearable sensors, like PPG, which would enable examining the associations at the intra-individual level in people with MCI. In this pilot experiment, we address these gaps by investigating the feasibility of using physiological measures based on wearable sensor data for predicting cognitive function in individuals with MCI. Using longitudinal dataset with repeated cognitive measures and physiological signals collected over 10 weeks, we examined the associations between digital physiological features and cognitive performance. We further evaluated the predictive ability of these digital physiological features to track inter- and intra-individual changes in cognitive function using machine learning methods. So far, no research has examined longitudinal associations between cognitive performance and physiological measures collected with a wearable device, and this is the first study that analyzed how changes in physiological measures over time are associated with concurrent changes in cognitive functions.

## Methods

### Data and participants

The data used in this work were obtained from the registered single-arm clinical trial (ClinicalTrials.gov, Identifier: NCT05059353) with multidomain clinical intervention conducted in Singapore from November 2021 to August 2022 by the National Neuroscience Institute. The digitally delivered intervention targeted to improve cognitive functions was provided to older adults (50–70 years old) diagnosed with amnestic MCI (*N* = 30) and age-matched cognitively normal individuals (*N* = 10) and lasted for a period of 10 weeks. For the purposes of this study, we used data only from participants with MCI as the expected variation in the cognitive performance is larger in this target group compared to cognitively normal participants. During the intervention, participants were provided with Empatica-E4, a medical-grade sensor-equipped wrist device that collects physiological signals, and instructed to wear it during therapy sessions, sleep, and as much as possible otherwise, however, as per the trial protocol, participants were not required to wear it around the clock. Inclusion/exclusion criteria is listed in the Table 1; other details of the intervention can be found in the clinical trial registry [27] and in the previous research [28].

**Table 1:** Inclusion/exclusion criteria for participants with MCI

Inclusion criteria	Exclusion criteria
Either male or female aged between 50 and 70 years (inclusive) Diagnosis of amnestic MCI using the Petersen’s criteria and/or the NIA-AA criteria Clinical Dementia Rating score of 0.5 and Mini-Mental State Examination > 24 Education > 6 years Literate in English Basic proficiency in using web-based applications/mobile platforms Willing to give informed consent	Significant hearing or visual impairment Significant systemic, neurological or psychiatric illness such as end stage renal failure, Parkinson’s disease or major depression Participation in any pharmacological or non-pharmacological (interventional) clinical trial in the preceding 12 weeks

### Target outcomes

All participants were given cognitive assessments at two timepoints—at baseline a week before the intervention began, and within a week after the 10-week intervention. The Neuropsychological Test Battery (NTB) was used to assess cognitive function [29]. Each test score from an individual was converted into a *z*-score using available normative data from test manuals stratified by age range and education level. Composite scores were calculated by averaging the *z*-scores across tests and the global score was calculated as a mean of all tests’ *z*-scores:

1. Executive Function: WAIS-IV Digit Span Task; Similarities; Trail-Making Test B; Category, Letter, and Category Switching Verbal Fluency tests.
2. Processing Speed: Trail-Making Test A; Symbol Digit Modalities Task
3. Immediate Memory: Logical Memory I, RAVLT trials I-V, and Visual Reproduction I.
4. Delayed Memory: Logical Memory II, RAVLT trial VII, and Visual Reproduction II.
5. Global Cognition was calculated from all the listed tests.

### Wearable signal processing and feature extraction

The E4 wristband collects four types of signals: (1) triaxial acceleration [gravitational units, g] with a sampling rate of 32 Hz, (2) skin temperature [°C]—4 Hz, (3) EDA or skin conductance [micro-Siemens]—4 Hz, and (4) blood volume pulse (BVP) with photoplethysmography sensor (PPG) [nanowatt]—64 Hz. Beyond the raw data available from the device, it also provides inter-beat-intervals (IBI) [seconds] and heart rate (HR) [beats per minute] data computed by the manufacturer’s algorithms from the same raw signals.

**Fig. 1:**
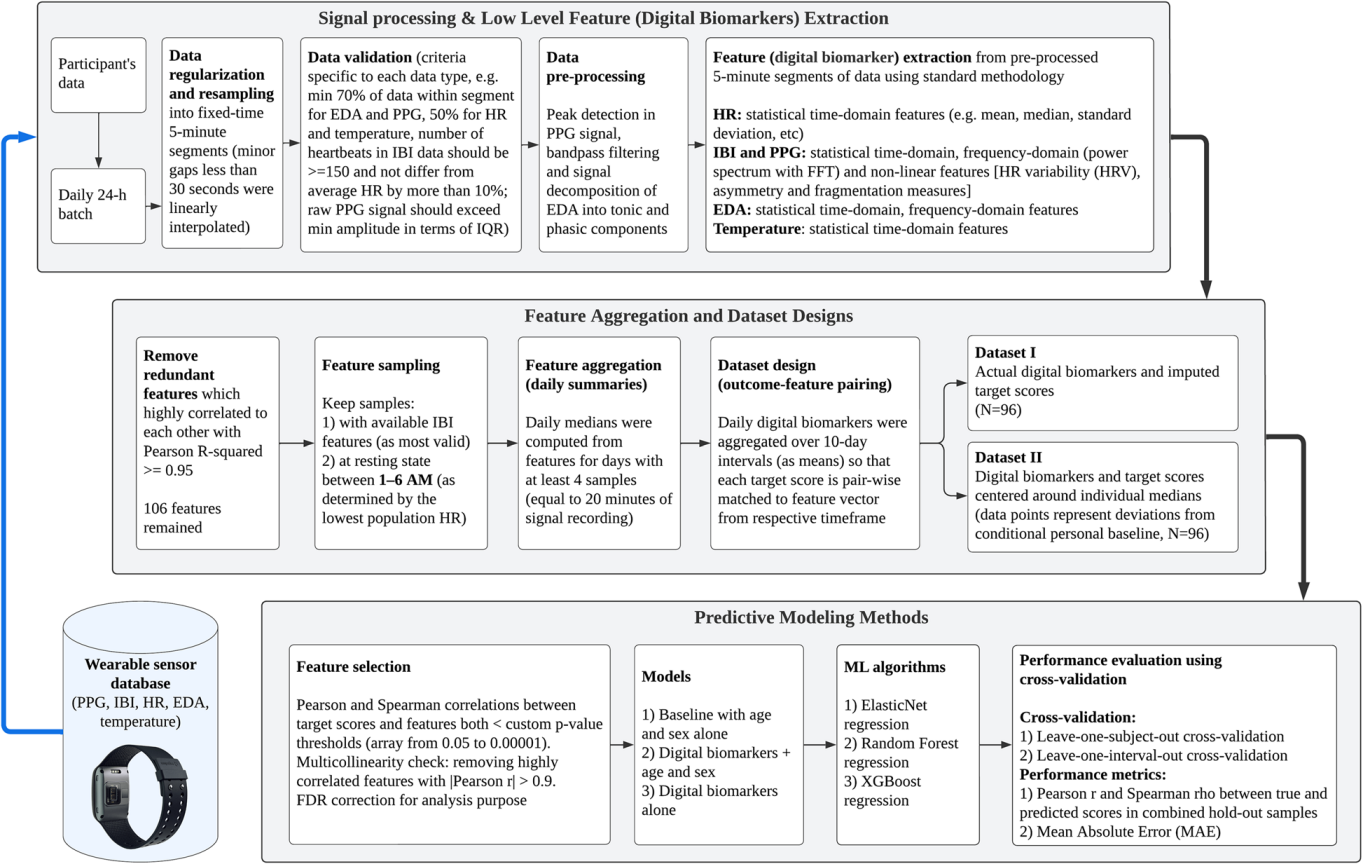
Data processing, feature extraction and predictive modeling pipeline

Wearable data were processed in daily batches. Each day-length signal was regularized and resampled into fixed 5-min segments from 00:00:00 to 23:59:59, as this is shortest recommended duration for HRV measurement [30] and minor gaps less than 30 s were linearly interpolated. Then samples with sufficient data (50% for HR and temperature, and 70% for EDA and PPG, while number of heartbeats in IBI could not differ from average HR in the same segment by more than 10%) were selected for further processing that was specific for each type of signal. Signal processing and feature extraction from wearable sensor data were performed in Python (version 3.9) using NeuroKit2 package (version 0.2.0) [31]; the entire pipeline is shown in Fig. 1. For example, the EDA signal went through a Butterworth bandpass filter and then was decomposed into tonic and phasic components, while blood volume pulse signal from the PPG sensor was processed with the Elgendi peak detection algorithm [32] to find heartbeats. Features were then computed from pre-processed samples using standard methodology including statistical time-domain (e.g., mean, median, standard deviation) and frequency-domain methods (power spectrum density estimation with fast Fourier transform). The following groups of features were computed: phasic skin conductance, tonic skin conductance (skin conductance responses), EDA frequency-domain indexes of sympathetic activation, time-domain, frequency-domain (from both beat-to-beat intervals and derived periodic HR signal) and non-linear HRV measures derived from the Poincaré plot, including measures of heart rate asymmetry and fragmentation [33] and based on both IBI data and PPG-based heartbeats, temperature time-domain measures. After computing all the features for each 5-min segment (the complete list of features summarized in Additional file 1: Table S1), we removed samples with unavailable IBI measures because it is considered most sensitive to inappropriate device use or non-use. Next, redundant measures highly correlated to each other (squared Pearson correlation coefficient ≥ 0.95) were removed and the remaining non-redundant measures were kept for further analysis. Since physiological measurements are recommended to be taken during a resting state [30], only samples from the calmest 5-h night-time window (from 1 to 6AM, as determined by the lowest rolling population mean HR) were kept for analysis. Finally, daily summary measures (medians) were computed from 5-min physiological features for days with at least four valid 5-min samples. In total, 106 features were used in further analysis, including 45 PPG-based features, 37 IBI-based, 12 EDA-based, 7 HR-based, and 5 temperature-based.

### Design of statistical analysis and predictive modeling

A longitudinal design was used to examine the dynamic changes over time and correlations between the two factors at the intra-individual level which provides stronger evidence for such relations than cross-sectional designs. NTB composite scores were only obtained at baseline and post-intervention. To show the feasibility of computing scores on a regular short-term basis and to maximally leverage available wearable data collected during the entire observation period, NTB composite scores were imputed to obtain datapoints within each interval matching respective samples of wearable data. We assumed that changes in cognitive performance across a short-term 10-week period were likely to occur monotonically and smoothly rather than with bidirectional oscillations, and changes tended to plateau off following a non-linear sigmoid function [34, 35]. Based on these assumptions, NTB composite scores for each 10-day interval (resulting in 7 repeated measurements per participant in total) were imputed using interpolation with a Gompertz curve (details on the function parameters are provided in the Additional file 1: Appendix S1). Respectively, daily digital physiological features were aggregated into means over the same 10-day intervals. To ensure the suggested imputation method did not introduce bias and significantly distort the data, we compared correlation coefficients observed in datasets with and without imputation (using paired *t*-test and Pearson correlation). We also compared it to two other methods (linear interpolation and random imputation) to check how interpolation methods affected observed correlations (see Additional file 1: Appendix S2 and Figures S1-S3 for more details).

Since between-individual differences in both cognitive measures and physiological parameters are likely to exceed within-individual differences occurring in the short term, the model based on such clustered data should be more accurate in determining an overall individual’s level of cognitive functioning relative to other people rather than sensing and predicting more subtle intra-individual changes. Hence, to address this limitation and mitigate differences between participants, the physiological measures were centered around individual median values representing deviations from conditional personal baseline, and NTB composite scores were centered around individual half-range values (i.e. median between original baseline and post-intervention scores). As a result, centering data allows us to examine whether intra-individual changes in physiological measures are associated with intra-individual changes in cognitive measures. Thus, two datasets for analysis were used: the first one with imputed NTB scores and actual physiological measures for testing the predictive ability of digital physiological features to determine the actual NTB scores, and the second one with individually centered physiological measures and NTB scores for evaluating the predictive ability to capture and track intra-individual changes in cognitive functioning.

Pearson and Spearman correlations were used to examine associations between physiological measures and cognitive functioning in both datasets. In addition, we used linear mixed-effect regression (LMER) models to test associations (fixed effects) between cognitive measures and digital physiological features as the data is grouped by participants. *P*-values were adjusted using Benjamini–Hochberg false discovery rate (FDR) correction across all outcome-feature pairs in all statistical analyses. Next, a series of supervised machine learning models predicting NTB scores were trained to evaluate the predictive ability of digital physiological features. Candidate features were selected using a statistical filter based on Pearson and Spearman correlations with varying levels of *p*-value significance ranging from < 0.05 to < 0.00001 (both correlation *p*-values should pass a threshold for selection). We also removed features highly correlated to each other (with absolute Pearson *r* > 0.9) to mitigate multicollinearity. Since age and sex [36] can impact both cognitive functioning and physiology, the added predictive value of digital physiological measures should be estimated relative to predictions based on demographics alone, and therefore, we trained and evaluated three separate models: a baseline model based only on age and sex, a combined model based on demographics and digital physiological features, and a model based on physiological features alone. Several machine learning algorithms were used for model training including elastic net linear regression, random forest regression, and extreme gradient boosting. In sum, for predicting each outcome we trained 12 different models (four *p*-value criteria for feature selection and three training algorithms). For performance evaluation of the predictive models, we used leave-one-subject-out and leave-one-interval-out cross-validation strategies, similar to the previous research [37]. In subject-based cross-validation all observations belonging to one subject were held for a test set and remaining data were used for training, while in interval-based cross-validation all observations from a particular interval were held for a test set and remaining data were used for training. These split-samplings were iteratively repeated until we obtained predictions for all subjects and intervals. In practice, the first scenario reflects making predictions for new never-seen subjects, while the second scenario reflects making predictions for existing subjects who have already been monitored for some time. Performance metrics were computed after all predictions obtained and included Pearson and Spearman correlation coefficients and mean absolute error (MAE) between actual and predicted values. Models with the highest Pearson *r* were considered the best. All statistical analysis and predictive modeling are done using Python (version 3.9) and numpy, pandas, scipy.stats, scikit-learn, XGBoost, and statsmodels packages.

## Results

### Characteristics of the data and participants

Twenty-seven MCI individuals out of the 30 recruited completed the clinical trial including the required neuropsychological assessments (one participant dropped out, one had low compliance to the therapy program, and another did not complete post-intervention assessment). We collected and processed 99,261 5-min samples of wearable data in total covering 1134 days of observation. Most missing data were due to participant non-compliance (e.g. non-wearing the device at night) or inappropriate device use (non-removing a USB charging dock which blocks sensors). Besides, data collection might be interrupted due to natural causes during normal daily use of the device, such as vibration and loss of contact between sensors and skin surface. After data cleaning and filtering, the total number of valid wearable data samples was 18,546 (or 92,730 min of data) covering 585 days across 17 subjects (34.4 days per subject on average), which were then aggregated by 10-day intervals. The final dataset included 96 aggregated intervals pairing NTB scores and digital physiological features. Further details of participants included into the analysis are shown in the Table 2.

**Table 2:** Characteristics of participants and data

Characteristic	Metric
Participants, *N*	17
Age, mean (standard deviation [SD])	60.3 (4.5)
Sex = female, *N* (*%*)	9 (52.9%)
Years of education, mean (SD)	13.5 (3.1)
*N* of valid wearable data samples per participant, mean (SD)	1093 (842.2)
*N* of valid wearable data samples per day, mean (SD)	31.7 (15.1)
*N* of days with available digital physiological features per participant, mean (SD)	34.4 (18.9)

Correlation coefficients between imputed Neuropsychological Test Battery (NTB) scores and digital physiological features were very similar to those observed in the dataset without imputation (for Pearson coefficients: *r* = 0.94, paired *t*-test *p*-value = 0.75; for Spearman coefficients: *r* = 0.94, paired *t*-test *p*-value = 0.29), demonstrating that datasets shared the same pattern of relationships, and the imputation did not introduce substantial bias. Moreover, comparison of different imputation methods demonstrated that the interpolation with Gompertz curve was almost identical to the linear interpolation in terms of similarity to each other and to the dataset without imputation, while it was significantly different from the random imputation where correlation coefficients were much closer to zero and different from the dataset without imputation (see Additional file 1: Figures S1-S3 for more details). Thus, the Gompertz curve provided an acceptable imputation which did not significantly affect the results (as random imputation did).

### Predicting NTB scores

**Fig. 2:**
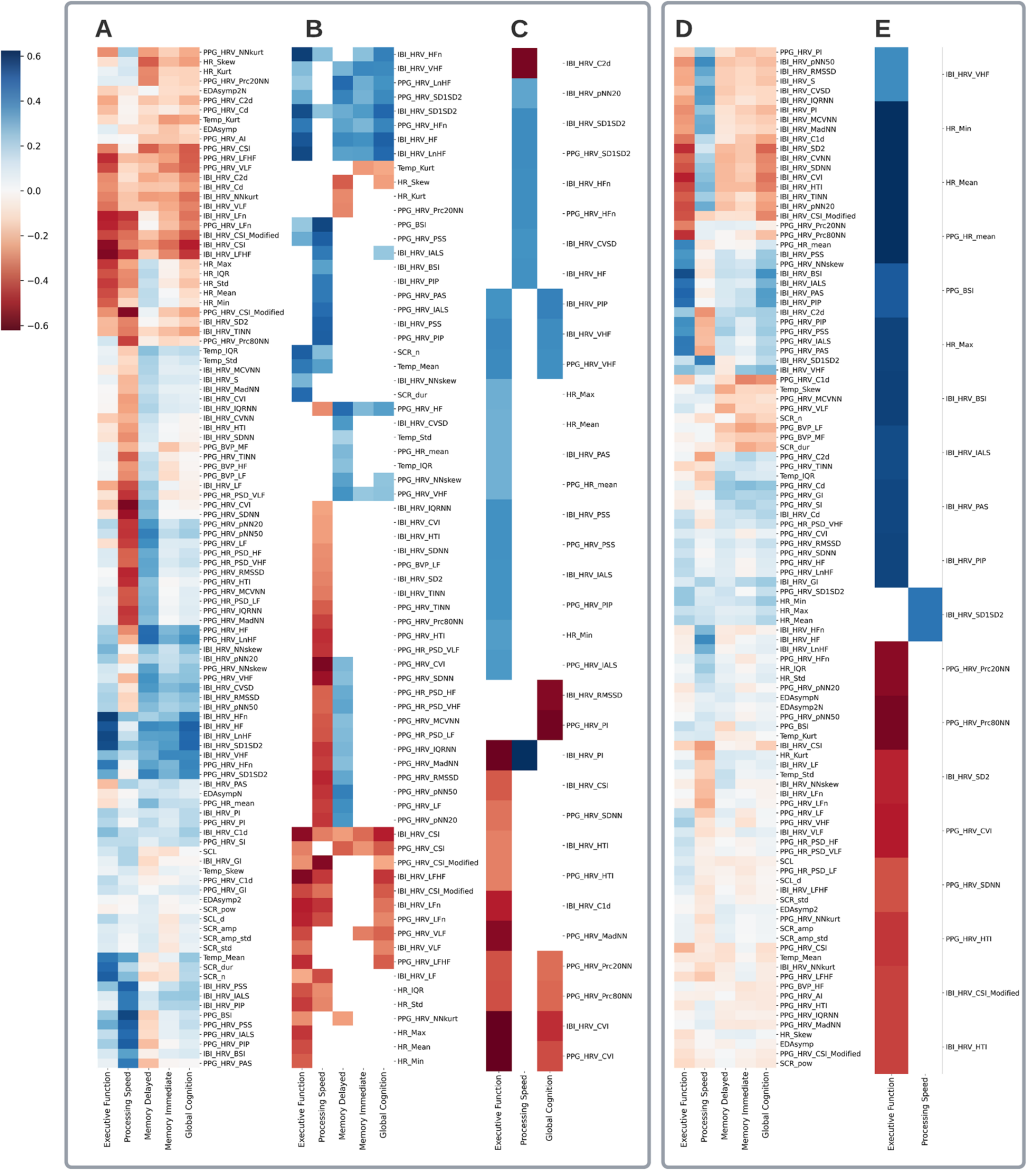
Correlation heatmaps between NTB composites and digital physiological features. Subplots **A–C** are based on dataset with actual NTB scores, **D,E**—on the centered dataset.** A** Color intensity is proportionate to the Pearson correlation coefficient, correlations between all outcome–feature pairs are shown. **B** Only significant correlations (both Spearman and Pearson adjusted *p*-values < 0.05 after the FDR correction) are displayed. **C** Color intensity is proportionate to the R^2^ of explained variance by fixed effect component from mixed-effect regression, only significant effects are shown (fixed effect *p*-values < 0.05 after FDR correction). **D** Color intensity is proportionate to the Pearson correlation coefficient, correlations between all outcome–feature pairs are shown. **E** Only significant correlations (both Spearman and Pearson adjusted *p*-values < 0.05 after FDR correction) are included

Digital physiological features had the greatest number of significant correlations with processing speed, executive function, and global cognition composites after the false discovery rate (FDR) correction—53, 44, and 39 out of 106 potential relations respectively (Fig. 2). On average, the strength of significant correlations was moderate, the mean absolute Pearson *r* is 0.46 (standard deviation [SD] 0.12) and Spearman rho was 0.45 (SD 0.11) for executive function, 0.37 (0.08) and 0.40 (0.07) for processing speed, 0.33 (0.06), 0.29 (0.04) for immediate memory, 0.36 (0.05) and 0.34 (0.05) for delayed memory, and 0.42 (0.08) and 0.37 (0.07) for global cognition. Processing speed had a distinct pattern of correlations different from other cognitive measures, i.e. it correlated to unique physiological measures which did not correlate to other cognitive measures.

In terms of specific digital physiological features, HRV measures (both photoplethysmography [PPG]- and inter-beat-intervals [IBI]-based) correlated with all cognitive composites, especially cardiac sympathetic index (CSI), logarithm of HRV-HF, normalized HRV-HF, the ratio of short-term to long-term variations in HRV (SD1SD2) (full results are in the Additional file 1: Tables S2-S4). Three temperature features correlated with delayed memory, and two EDA features—with executive function.

**Fig. 3:**
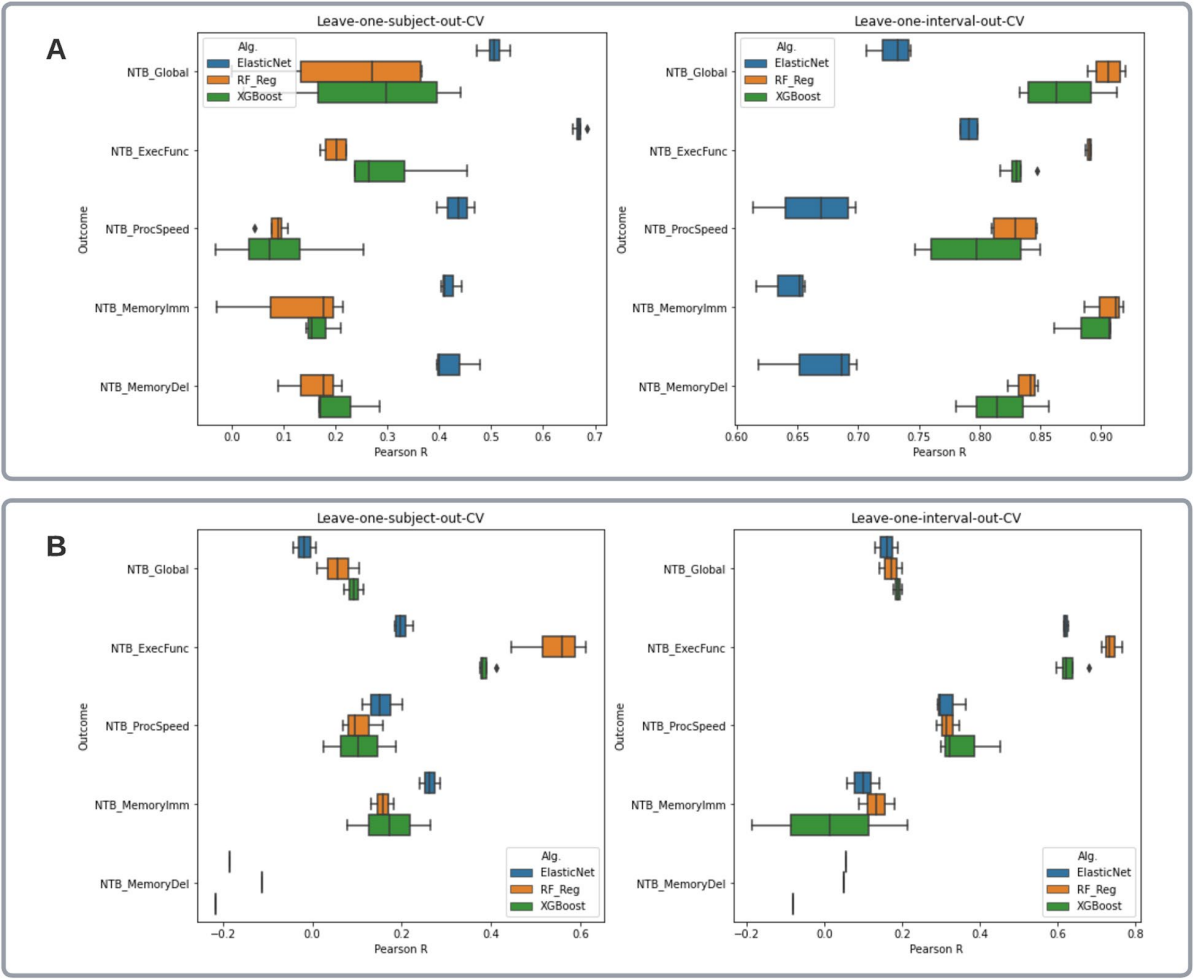
Boxplots of models’ performance (Pearson *r*) across outcomes, machine learning algorithms, and types of cross-validation; each box represents all performance values obtained with different feature sets depending on varied feature selection criteria. **A** Predicting actual NTB scores. **B** Predicting intra-individual changes in NTB scores from intra-individual changes in physiological features (centered dataset)

The performance of predictive models combining digital physiological features and demographics is shown in Tables 3 and 4 and in Fig. 3A. The best predictable outcome from digital physiological features and demographics in never-seen subjects was the executive function composite (*r* = 0.69, rho = 0.70, mean absolute error [MAE] = 0.46). The addition of digital physiological features brought large improvements in the prediction of executive function in terms of gained Spearman correlation between the actual and predicted scores, while global cognition and immediate memory were predicted with the same accuracy from age and sex alone. Greater gains in Spearman correlation than in Pearson between the actual and predicted scores indicated that the physiological features were useful in sensing and correctly detecting minor monotonic differences between individuals, which was not possible with age and sex alone. Elastic net linear regression outperformed other ML algorithms for all outcomes within the subject-based cross-validation, while tree-based algorithms were better with the interval-based cross-validation. Predictions with training and cross-validation by intervals were much more accurate than by subjects for all outcomes and range from 0.85 to 0.92 in terms of Pearson correlation and the added predictive value of physiological measures was also larger for all outcomes ranging from 0.22 to 0.38 in terms of improved correlation. Features used in each of the best models are listed in Additional file 1: Table S5.

**Table 3:** Performance of the best models predicting NTB scores

Cross-validation method	Cognitive measure	Machine Learning algorithm	Number of used features	Feature selection *p*-value	*r*	*r* gain	rho	rho gain	MAE
Subject-based	Global Cognition	ElasticNet	11	0.001	0.54	0.02	0.50	− 0.06	0.59
	Executive Function	ElasticNet	13	0.001	0.69	0.15	0.70	0.46	0.46
	Processing Speed	ElasticNet	14	0.001	0.47	0.08	0.48	0.27	0.67
	Memory Immediate	ElasticNet	4	0.001	0.44	− 0.03	0.44	0.01	1.24
	Memory Delayed	ElasticNet	6	0.001	0.48	0.22	0.61	0.33	0.97
Interval-based	Global Cognition	Random Forest	7	0.0001	0.92	0.25	0.91	0.25	0.16
	Executive Function	Random Forest	9	0.00001	0.89	0.22	0.87	0.33	0.15
	Processing Speed	XGBoost	11	0.0001	0.85	0.33	0.82	0.31	0.21
	Memory Immediate	Random Forest	3	0.0001	0.92	0.29	0.87	0.28	0.33
	Memory Delayed	XGBoost	3	0.0001	0.86	0.38	0.86	0.37	0.35

**Table 4:** Average models’ performance (Pearson *r*) in predicting NTB scores across varying feature selection criteria

Cross-validation method	Cognitive measure	Machine Learning algorithms		
		**ElasticNet**	**Random Forest**	**XGBoost**
Subject-based	Global Cognition	0.51	0.23	0.26
	Executive Function	0.67	0.20	0.31
	Processing Speed	0.43	0.08	0.09
	Memory Immediate	0.42	0.12	0.17
	Memory Delayed	0.42	0.16	0.21
Interval-based	Global Cognition	0.73	0.91	0.87
	Executive Function	0.79	0.89	0.83
	Processing Speed	0.66	0.83	0.80
	Memory Immediate	0.64	0.91	0.89
	Memory Delayed	0.67	0.84	0.82

### Predicting intra-individual changes in NTB scores

The correlations between intra-individual changes in digital physiological features and intra-individual changes in NTB scores (i.e. correlations in the centered dataset where each data value represents deviation from an individual median) were weaker than the correlations in the non-centered dataset where all values were on the unadjusted common scale. However, there were still several significant correlations with executive function (18 physiological features) and processing speed (one feature) sustained after the FDR correction (Fig. 2D, E). On average, the strengths of significant correlations were moderate, the mean absolute Pearson *r* was 0.55 (SD 0.09) and Spearman rho was 0.37 (SD 0.03) for executive function, and 0.47 and 0.33 for processing speed respectively. Only the changes in HR and HRV (both PPG- and IBI-based) measures were significantly associated with changes in cognitive composite scores, while EDA and temperature features became non-significant after the FDR correction. In addition, only three physiological features were consistently significant in the analysis of both datasets indicating the same relationship on between- and intra-individual levels, including negative correlation of IBI-based modified CSI and positive correlation of the power spectrum in the very high-frequency band of IBI (HRV-VHF) to executive function scores, and positive correlation of IBI-based SD1SD2 HRV index to processing speed scores. Mixed-effect regression also showed several significant effects between cognitive performance (executive function, processing speed and global cognition) and 34 physiological measures after the FDR correction confirming associations on the intra-individual level (Fig. 2C).

Performance of models predicting intra-individual changes in cognitive scores from changes in digital physiological features and demographics is shown in Table 5 and 6 and in Fig. 3B. Among the different NTB composites, intra-individual changes in executive function were best predicted—the best model was based on 13 digital physiological features (plus age and sex) and the random forest algorithm and performed with Pearson *r* = 0.61 (rho = 0.44, MAE = 0.07) in the individual-based cross-validation and *r* = 0.77 (rho = 0.48, MAE = 0.06) in the interval-based cross-validation. Intra-individual changes in other NTB scores were less predictable from physiological measures. Age and sex were not informative for predicting intra-individual changes in all outcomes (all predictions were constant scores), so the achieved performance was entirely due to digital physiological features and their interplay with demographics.

**Table 5:** Performance of the best models predicting intra-individual changes in NTB scores

Cross-validation method	Cognitive measure	Machine Learning algorithm	Number of used features	Feature selection *p*-value	*r*	*r* gain	rho	rho gain	MAE
Subject-based	Global Cognition	Random Forest	19	0.05	0.11	0.11	0.12	0.12	0.11
	Executive Function	Random Forest	15	0.001	0.61	0.61	0.44	0.44	0.07
	Processing Speed	ElasticNet	3	0.001	0.20	0.20	0.23	0.23	0.14
	Memory Immediate	ElasticNet	7	0.05	0.29	0.35	0.31	0.41	0.21
	Memory Delayed	Random Forest	6	0.05	− 0.11	− 0.11	− 0.05	− 0.05	0.19
Interval-based	Global Cognition	XGBoost	4	0.01	0.20	0.20	0.06	0.06	0.09
	Executive Function	Random Forest	15	0.001	0.77	0.77	0.48	0.48	0.06
	Processing Speed	XGBoost	3	0.001	0.45	0.45	0.30	0.30	0.14
	Memory Immediate	XGBoost	3	0.01	0.21	0.54	0.22	0.20	0.25
	Memory Delayed	XGBoost	6	0.05	0.06	0.06	− 0.01	− 0.01	0.17

**Table 6:** Average models’ performance (Pearson *r*) in predicting intra-individual changes in NTB scores across varying feature selection criteria

Cross-validation method	Cognitive measure	Machine Learning algorithms		
		**Elastic Net**	**Random Forest**	**XGBoost**
Subject-based	Global Cognition	− 0.02	0.06	0.09
	Executive Function	0.20	0.54	0.39
	Processing Speed	0.15	0.11	0.10
	Memory Immediate	0.26	0.16	0.17
	Memory Delayed	− 0.19	− 0.11	− 0.22
Interval-based	Global Cognition	0.16	0.17	0.19
	Executive Function	0.62	0.74	0.63
	Processing Speed	0.32	0.32	0.36
	Memory Immediate	0.10	0.13	0.01
	Memory Delayed	0.06	0.05	− 0.08

## Discussion

This study examined the feasibility of using digital physiological features from wearables as a proxy for measuring cognitive function. We analyzed the associations between physiological digital physiological features and NTB cognitive composite scores and evaluated the predictive ability of models, combining these physiological features with demographics, to determine scores across four cognitive domains (global, memory, executive function, and processing speed) and to track more subtle short-term intra-individual changes. Using longitudinal wearable data collected over the 10-week clinical trial, we showed that physiological measures, primarily HRV measures, had significant and strong associations with the executive function composite, but not for other composites. Among the models trained to predict executive function, the best model predicted scores in never-seen individuals with Pearson *r* = 0.69 (MAE = 0.46) and predicted intra-individual changes in these scores with *r* = 0.61 (MAE = 0.07). The accuracy of predicting executive function in the same individuals but for new time interval samples was notably higher, with Pearson *r* = 0.89 (MAE = 0.15) for actual scores and *r* = 0.77 (MAE = 0.06) for score changes. The models with digital physiological features consistently and substantially outperformed baseline models based on age and sex alone in predicting executive function, highlighting the added predictive value of digital physiological features. Other cognitive measures had weaker correlations with physiological measures and were predicted with lower accuracy, especially when predicting intra-individual changes.

To our knowledge, this is the first study with a longitudinal design that found that intra-individual changes in executive function score were associated with intra-individual changes in HRV measures, which characterize sympathovagal balance in the ANS. Unlike prior research that primarily examined the strength of associations [10–12], this study additionally evaluated the predictive ability of physiological parameters in determining cognitive test performance using machine learning methods and cross-validation. Furthermore, this study contributed to the existing body of evidence regarding associations between HRV measures and cognitive function in individuals with MCI [12].

While the data suggest that changes in cognitive function and changes in physiological parameters are related, the exact mechanisms underpinning these correlations remain to be elucidated. One possible explanation for the observed relationship between HRV measures and cognition is the disruption of the brain cholinergic system and acetylcholine deficiencies in Alzheimer’s disease, which then may result in downstream cognitive symptoms related to the disturbance of the ANS [38, 39]. Possibly, this may be due to changes in vagus nerve functions leading to changes in cognition [40, 41]. For example, the recent experimental study showed that stimulating vagus nerve and modulating heart rate oscillations via slow paced breathing intervention affect plasma amyloid beta and tau levels [42]. Furthermore, mediation analysis showed that autonomic dysfunction reflected in attenuated HRV measures during non-REM sleep contributed to complement activation and deposition of AD biomarkers leading to cognitive impairment [43]. In addition, reduced parasympathetic activity (lower HRV-HF) during deep sleep was associated with weaker functional connectivity within core and broader central-autonomic network brain regions in older adults at risk of dementia [44]. Finally, it is not clear why the correlations between digital physiological features and cognitive measures were stronger with executive function compared to other composites. Executive function tasks are often thought to draw on many other cognitive processes, such as perception, memory, and strategic planning [45], and, therefore, they may make variable energy demands and require optimal heart function for distribution of energizing oxygenated blood consistent with the neurovisceral integration model.

This study has several strengths. First, we used two datasets for analysis and predictive modeling: original un-centered and centered. The original dataset was used to analyze associations on the inter-individual level, whereas the centered dataset mitigated inter-individual differences and allowed to reveal whether intra-individual changes in physiological paraments (i.e. deviations from some conditional personal baseline) were associated with concurrent intra-individual changes in cognitive function. Second, we used two common cross-validation strategies to evaluate models’ accuracy. Although leave-one-interval-out-cross-validation is more prone to overfitting because cognitive scores from the same individuals tend to be similar across intervals, this method allowed us to better evaluate the added predictive value of physiological measures against demographic information. Leave-one-subject-out-cross-validation consistently showed lower accuracy, however it provided a more conservative and generalizable performance evaluation, as predictions were done in individuals that the models were not trained on. Thus, performance measures obtained with the two approaches provided a comprehensive evaluation of the predictive value of digital physiological features in monitoring cognitive functioning. Finally, we leveraged physiological signals collected during nocturnal sleep, which demonstrated their utility in studying brain–heart interaction in people with MCI. This allowed us to analyze autonomic function in isolation from psychological and behavioral confounders related to wakeful activity.

The main limitation of the study is its small sample size (N = 96 observations from 17 participants) and, therefore, the models should be verified with another independent sample. Also, a larger sample size would allow using deep learning methods to reveal complex non-linear patterns in the data. Another limitation is a relatively short period of the study observation (3 months) which restricts capturing a greater and long-term variation in cognition. In addition, the study sample is homogeneous and included mainly participants of Singaporean Chinese ethnicity. Thus, it is possible the results would not generalize to other populations. Another limitation stems from the study design since it was based on a multidomain intervention, which might confound some associations by causing concurrent changes in these factors independently. Consistent with previous literature, yoga exercises and meditation provided as part of the intervention could induce changes in HRV [46, 47] and affect cognition [48, 49], while cognitive games and reminiscence therapy may have contributed to changes in cognition. However, executive function, processing speed and delayed memory changed independently and sometimes in opposing directions (increased or decreased) in the subsample of the trial participants included in the analysis (see Additional file 1: Figure S4 and Table S6 for more details). In addition, there was no significant increase in executive function from baseline to post-intervention. Therefore, the intervention likely did not falsify the correlation between the physiological parameters and cognitive measures.

Next, since most analyzable data were collected while participants slept, we were unable to use acceleration data capturing physical gross-motor activity which could be informative for detecting cognitive impairment [50, 51]. Similarly, due to the lack of day-time data we were unable to examine circadian and rest-activity rhythms which are also associated with changes in cognitive function [52–54]. Another limitation is using imputed target outcomes data (NTB scores) for model training and testing instead of real weekly measurements. To mitigate this limitation, we compared different interpolation methods and demonstrated that correlation coefficients between NTB scores and digital physiological features were very similar to those obtained without imputation that indicated identical relationships pattern between datasets. In addition, the range of cognitive scores of the participants was limited, and it is unclear whether the trained models would be able to predict over clinically significant range of scores. Finally, despite a range of reliable and moderately strong associations, the achieved accuracy of predictive models is still below the desirable level, and hence the wearable-based physiological measures are not yet able to replace standardized cognitive tests.

To overcome these limitations, we recommend that future research leverage a larger and preferably multi-ethnic (multicenter) sample to achieve greater statistical power and generalizability, a longer follow-up duration (6 or 12 months) to capture greater variation in cognition, and digital cognitive assessments in addition to NTB for more frequent outcomes measurement. Finally, future research should leverage data from a group not receiving the intervention, either through a pure longitudinal observational study or by utilizing a control group in the case of a clinical trial.

## Conclusions

In conclusion, our findings suggest that physiological measures correlate to cognitive function in individuals with MCI. Therefore, these physiological features have potential to be used for passive assessment of cognitive function using wearable sensors in real time and in ambulatory settings. In turn, continuous monitoring of cognitive function in individuals with MCI would allow a better understanding of the response to treatment and the delivery of more effective personalized therapies. The most informative and valuable physiological features were HRV features measuring sympathovagal balance in ANS, that is in line with the neurovisceral integration model of central-autonomic neural network. The results from this study are promising and warrant further research which should validate these physiological measures and evaluate their value when combined with other digital phenotyping data such as speech, language use, software interaction, finger dexterity, as well as demographic and medical history.

### Supplementary Information

**Additional file 1: Table S1.** Digital physiological features. **Appendix S1.** Gompertz function parameters. **Appendix S2.** Comparison of imputation methods. **Figures S1-S3.** Comparison of correlation coefficients between NTB composite scores and digital physiological features in datasets with and without imputation and using different interpolation methods. **Table S2.** Spearman and Pearson correlations between absolute NTB composite scores and physiological features. **Table S3.** Spearman and Pearson correlations between intra-individual changes in NTB composite scores and intra-individual changes in physiological features. **Table S4.** Linear mixed-effects regression results. **Table S5.** Features used in the best models predicting NTB composite scores. **Figure S4.** NTB composite scores: changes from baseline to post-intervention. **Table S6.** Mean and standard deviation of NTB composite scores at the baseline and post-intervention assessments.

## Data Availability

The datasets generated and/or analyzed during the current study are not publicly available due to their containing information that could compromise the privacy of research participants but are available via mediated access from the corresponding author with the permission of Neuroglee Therapeutics on reasonable request. To protect privacy of the research participants a requester must sign a document indicating they will not link the data with any other datasets, and the researcher must have received recognized training on human research ethics.

## Abbreviations

AD: Alzheimer’s disease
ANS: Autonomic nervous system
CSI: Cardiac Sympathetic Index
EDA: Electrodermal activity
FDR: False discovery rate
HR: Heart rate
HRV: Heart rate variability
HRV-HF: Heart rate variability – high frequency
HRV-VHF: Heart rate variability – very high frequency
IBI: Inter-beat interval
LMER: Linear mixed-effect regression
MAE: Mean absolute error
MCI: Mild cognitive impairment
ML: Machine learning
NTB: Neuropsychological Test Battery
PPG: Photoplethysmography
RAVLT: Rey Auditory Verbal Learning Test
RMSSD: Root mean of the square successive differences
RSA: Respiratory sinus arrhythmia
WAIS-IV: Weschler Adult Intelligence Scale 4th Edition
