# Supplementary material for: Predicting cognitive scores from wearable-based digital physiological features using machine learning: data from a clinical trial in mild cognitive impairment
Source: BMC Med. 2024 Jan 25;22:36. doi: 10.1186/s12916-024-03252-y (PMC10809621; doi:10.1186/s12916-024-03252-y)
Supplement: Supplementary file 1 — Additional file 1: Table S1. Digital physiological features. Appendix S1. Gompertz function parameters. Appendix S2. Comparison of imputation methods. Figures S1-S3. Comparison of correlation coefficients between NTB composite scores and digital physiological features in datasets with and without imputation and using different interpolation methods. Table S2. Spearman and Pearson correlations between absolute NTB composite scores and physiological features. Table S3. Spearman and Pearson correlations between intra-individual changes in NTB composite scores and intra-individual changes in physiological features. Table S4. Linear mixed-effects regression results. Table S5. Features used in the best models predicting NTB composite scores. Figure S4. NTB composite scores: changes from baseline to post-intervention. Table S6. Mean and standard deviation of NTB composite scores at the baseline and post-intervention assessments. [file 12916_2024_3252_MOESM1_ESM.docx]

Additional file 1

For the manuscript “Predicting Cognitive Scores from Wearable-based Digital Physiological Features Using Machine Learning: Data from a Clinical Trial in Mild Cognitive Impairment” authored by Yuri G. Rykov, Michael D. Patterson, Bikram A. Gangwar, Syaheed B. Jabar, Jacklyn Leonardo, Kok Pin Ng, and Nagaendran Kandiah

**Table S1. Digital physiological features**

| Sensor data | Category | Digital physiological feature (5-minute segment) | Definition |
| --- | --- | --- | --- |
| Electrodermal activity (EDA), microSiemens [4Hz] | Phasic skin conductance | SCL | Skin conductance level, mean value of tonic skin conductance |
|  |  | SCL_IQR | Interquartile range of tonic skin conductance |
|  | Tonic skin conductance (skin conductance responses) | SCR_std | Standard deviation (Std) of phasic skin conductance |
|  |  | SCR_pow | Power of phasic skin conductance [mean squared value] |
|  |  | SCR_n | Number of skin conductance responses [i.e peaks in phasic skin conductance] |
|  |  | SCR_rate | Rate of skin conductance responses [N responses per sec] |
|  |  | SCR_amp | Mean amplitude of phasic skin conductance (responses) |
|  |  | SCR_amp_std | Std of amplitudes of phasic skin conductance (responses) |
|  |  | SCR_dur | Skin conductance responses duration (sum of rise times) |
|  | EDA frequency-domain indexes of sympathetic nervous system | EDAsymp | Index of sympathetic control based on the power spectral analysis of EDA, power within 0.045 to 0.25 Hz frequency band of EDA signal ("ghiasi" method) |
|  |  | EDAsympN | Normalized index of sympathetic control ("ghiasi" method) [obtained by dividing EDAsymp by total power] |
|  |  | EDAsymp2 | Index of sympathetic control based on the power spectral analysis of EDA, power within 0.045 to 0.25 Hz frequency band of EDA signal ("posada" method) |
|  |  | EDAsymp2N | Normalized index of sympathetic control ("posada" method) [obtained by dividing EDAsymp by max power] |
| Blood volume pulse (BVP) based on photoplethysmography (PPG), nanowatts [64Hz]  Inter-beat interval (IBI) data based on PPG (provided by device and derived with manufacturer's algorithms) | Heart Rate Variability (HRV) time-domain measures (based either on RRI derived from raw PPG or on manufacturer-derived IBI) | [PPG/IBI]_HRV_RMSSD | Root Mean Square of Successive Differences of R-R intervals (RRI) (or Inter Beat Intervals) |
|  |  | [PPG/IBI]_HRV_SDNN | Std of RRIs (IBIs) |
|  |  | [PPG/IBI]_HRV_SDSD | Std of successive differences between RRIs |
|  |  | [PPG/IBI]_HRV_CVNN | Coefficient of variation of RRIs (IBIs) [Std divided by mean] |
|  |  | [PPG/IBI]_HRV_CVSD | HRV_RMSSD divided by MeanNN |
|  |  | [PPG/IBI]_HRV_MadNN | Median absolute deviation of the RR intervals. |
|  |  | [PPG/IBI]_HRV_MCVNN | The median absolute deviation of the RR intervals (MadNN) divided by the median of the RR intervals (MedianNN) |
|  |  | [PPG/IBI]_HRV_IQRNN | The interquartile range (IQR) of the RR intervals |
|  |  | [PPG/IBI]_HRV_pNN50 | The proportion of RR intervals greater than 50ms, out of the total number of RR intervals |
|  |  | [PPG/IBI]_HRV_pNN20 | The proportion of RR intervals greater than 20ms, out of the total number of RR intervals |
|  |  | [PPG/IBI]_HRV_Prc20NN | The 20th percentile of the RRIs |
|  |  | [PPG/IBI]_HRV_Prc80NN | The 80th percentile of the RRIs |
|  |  | [PPG/IBI]_HRV_NNkurt | Kurtosis of RRI distribution |
|  |  | [PPG/IBI]_HRV_NNskew | Skewedness of RRI distribution |
|  |  | [PPG/IBI]_Mean_HR | Mean HR |
|  |  | [PPG/IBI]_HRV_TINN | A geometrical parameter of the HRV, or more specifically, the baseline width of the RR intervals distribution obtained by triangular interpolation, where the error of least squares determines the triangle. It is an approximation of the RR interval distribution |
|  |  | [PPG/IBI]_HRV_HTI | The HRV triangular index, measuring the total number of RR intervals divided by the height of the RR intervals histogram |
|  |  | [PPG/IBI]_BSI | Baevsky Stress Index (width-to-hight ratio of a RRI histogram) |
|  | HRV frequency-domain measures (based either on RRI derived from raw PPG or on manufacturer-derived IBI) | [PPG/IBI]_HRV_VLF | PSD in very low freqyency band [.0033 to .04 Hz] |
|  |  | [PPG/IBI]_HRV_LF | PSD in low freqyency band [.04 to .15 Hz] |
|  |  | [PPG/IBI]_HRV_HF | PSD in high freqyency band [.15 to .4 Hz] |
|  |  | [PPG/IBI]_HRV_VHF | PSD in very high freqyency band [.4 to .5 Hz] |
|  |  | [PPG/IBI]_HRV_LFHF | Ratio of power in low frequency to power in high frequency |
|  |  | [PPG/IBI]_HRV_LFn | The normalized low frequency, obtained by dividing the low frequency power by the total power |
|  |  | [PPG/IBI]_HRV_HFn | The normalized high frequency, obtained by dividing the low frequency power by the total power |
|  |  | [PPG/IBI]_HRV_LnHF | The log transformed HF |
|  | HRV nonlinear measures – Poincaré plot geometry features (based either on RRI derived from raw PPG or on manufacturer-derived IBI) | [PPG/IBI]_HRV_SD1 | Standard deviation perpendicular to the line of identity. It is an index of short-term RR interval fluctuations, i.e., beat-to-beat variability. It is equivalent (although on another scale) to RMSSD |
|  |  | [PPG/IBI]_HRV_SD2 | Standard deviation along the identity line. Index of long-term HRV changes |
|  |  | [PPG/IBI]_HRV_SD1SD2 | ratio of SD1 to SD2. Describes the ratio of short term to long term variations in HRV |
|  |  | [PPG/IBI]_HRV_S | Area of ellipse described by SD1 and SD2 [pi * SD1 * SD2] |
|  |  | [PPG/IBI]_HRV_CSI | The Cardiac Sympathetic Index (Toichi, 1997) is a measure of cardiac sympathetic function independent of vagal activity, calculated by dividing the longitudinal variability of the Poincaré plot (4*SD2) by its transverse variability (4*SD1) |
|  |  | [PPG/IBI]_HRV_CVI | The Cardiac Vagal Index (Toichi, 1997) is an index of cardiac parasympathetic function (vagal activity unaffected by sympathetic activity), and is equal to the logarithm of the product of longitudinal (4*SD2) and transverse variability (4*SD1) |
|  |  | [PPG/IBI]_HRV_CSI_Mod | The modified CSI (Jeppesen, 2014) obtained by dividing the square of the longitudinal variability by its transverse variability |
|  | HRV measures of heart rate fragmentation (based either on RRI derived from raw PPG or on manufacturer-derived IBI) | [PPG/IBI]_HRV_PIP | Percentage of inflection points of the RR intervals series |
|  |  | [PPG/IBI]_HRV_IALS | Inverse of the average length of the acceleration/deceleration segments |
|  |  | [PPG/IBI]_HRV_PSS | Percentage of short segments |
|  |  | [PPG/IBI]_HRV_PAS | Percentage of NN intervals in alternation segments |
|  | HRV measures of heart rate asymmetry – asymmetry of the Poincaré plot (based either on RRI derived from raw PPG or on manufacturer-derived IBI) | [PPG/IBI]_HRV_GI | Guzik’s Index, defined as the distance of points above line of identity (LI) to LI divided by the distance of all points in Poincaré plot to LI except those that are located on LI |
|  |  | [PPG/IBI]_HRV_SI | Slope Index, defined as the phase angle of points above LI divided by the phase angle of all points in Poincaré plot except those that are located on LI |
|  |  | [PPG/IBI]_HRV_AI | Area Index, defined as the cumulative area of the sectors corresponding to the points that are located above LI divided by the cumulative area of sectors corresponding to all points in the Poincaré plot except those that are located on LI |
|  |  | [PPG/IBI]_HRV_PI | Porta’s Index, defined as the number of points below LI divided by the total number of points in Poincaré plot except those that are located on LI |
|  |  | [PPG/IBI]_HRV_C1d | Contributions of heart rate decelerations to short-term HRV (Piskorski, 2011) |
|  |  | [PPG/IBI]_HRV_C1a | Contributions of heart rate accelerations to short-term HRV (Piskorski, 2011) |
|  |  | [PPG/IBI]_HRV_SD1d | Short-term variance of contributions of decelerations (prolongations of RR intervals) (Piskorski, 2011) |
|  |  | [PPG/IBI]_HRV_SD1a | Short-term variance of contributions of accelerations (shortenings of RR intervals) (Piskorski, 2011) |
|  |  | [PPG/IBI]_HRV_C2d | Contributions of heart rate decelerations to long-term HRV (Piskorski, 2011) |
|  |  | [PPG/IBI]_HRV_C2a | Contributions of heart rate accelerations to long-term HRV (Piskorski, 2011) |
|  |  | [PPG/IBI]_HRV_SD2d | Long-term variance of contributions of decelerations (prolongations of RR intervals) (Piskorski, 2011) |
|  |  | [PPG/IBI]_HRV_SD2a | Long-term variance of contributions of accelerations (shortenings of RR intervals) (Piskorski, 2011) |
|  |  | [PPG/IBI]_HRV_Cd | Total contributions of heart rate decelerations to HRV |
|  |  | [PPG/IBI]_HRV_Ca | Total contributions of heart rate accelerations to HRV |
|  |  | [PPG/IBI]_HRV_SDNNd | Total variance of contributions of decelerations (prolongations of RR intervals) (Piskorski, 2011). |
|  |  | [PPG/IBI]_HRV_SDNNa | Total variance of contributions of accelerations (shortenings of RR intervals) (Piskorski, 2011). |
| PPG-based BVP signal | HRV frequency-domain measures based on derived and regularized (periodic) HR signal | PPG_HR_PSD_VLF | PSD based on periodic (regularly spaced) HR signal in very low freqyency band [.0033 to .04 Hz] |
|  |  | PPG_HR_PSD_LF | PSD based on periodic (regularly spaced) HR signal in low freqyency band [.04 to .15 Hz] |
|  |  | PPG_HR_PSD_HF | PSD based on periodic (regularly spaced) HR signal in high freqyency band [.15 to .4 Hz] |
|  |  | PPG_HR_PSD_VHF | PSD based on periodic (regularly spaced) HR signal in very high freqyency band [.4 to .5 Hz] |
|  | Raw BVP frequency-domain measures | PPG_BVP_LF | PSD of raw BVP signal in low freq band 0.05-0.70Hz |
|  |  | PPG_BVP_MF | PSD of raw BVP signal in middle freq band 0.70-2.00Hz |
|  |  | PPG_BVP_HF | PSD of raw BVP signal in high freq band 2.00-10.00Hz |
| HR data (beats per minute, bpm) (provided as derived by manufacturer algorithms) | Heart Rate (HR) | HR_IQR | IQR of HR |
|  |  | HR_Kurt | Kurtosis of HR distribution |
|  |  | HR_Max | Max HR |
|  |  | HR_Mean | Mean HR |
|  |  | HR_Median | Median HR |
|  |  | HR_Min | Min HR |
|  |  | HR_Skew | Skewedness of HR distribution |
|  |  | HR_Std | Std of HR |
| Skin temperature, °C  [4Hz] | Raw temperature time-domain measures | Temp_IQR | IQR of temperature |
|  |  | Temp_Kurt | Kurtosis of temperature distribution |
|  |  | Temp_Max | Max temperature |
|  |  | Temp_Mean | Mean temperature |
|  |  | Temp_Median | Median temperature |
|  |  | Temp_Min | Min temperature |
|  |  | Temp_Skew | Skewedness of temperature distribution |
|  |  | Temp_Std | Std of temperature |

**Appendix S1. Gompertz function parameters**

Gompertz function equation:


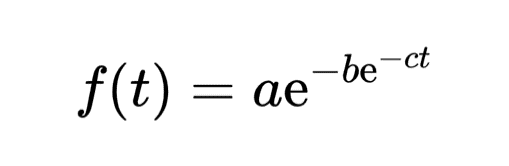


We assumed:
*b* (a displacement parameter) is constant (b=1.25),

*a* (an asymptote) is based on the difference between post-intervention and baseline scores,

*c* (growth rate) is scaled on *a* parameter, so that *c* is higher if the magnitude of change is greater, and *c* is lower (and hence interpolation is closer to linear) if the magnitude of change is smaller, as follows:

*c* = *c*_mean_ + ½ (*c*_norm_ - 0.5)

where *c*_mean_ = 0.7 (growth rate for average absolute *a*),

*c*_norm_ is a cumulative density function (CDF) based on z-score of observed absolute *a*

**Appendix S2.** Comparison of imputation methods

Comparison of correlation coefficients between NTB composite scores and digital physiological features in datasets with and without imputation and using different interpolation methods (linear and random). Random interpolation was done using study sample-based mean and standard deviations of respective NTB composites. Points on scatterplots represent outcome–feature pairs with respective correlation coefficients.

**Figure S1.** Comparison of Pearson and Spearman correlations coefficients between datasets with imputation (based on the Gompertz curve) and without imputation.


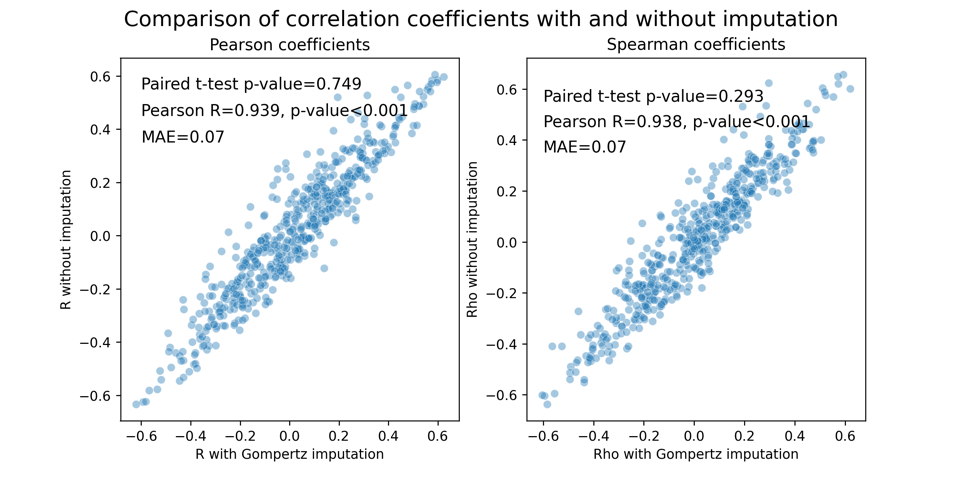


**Figure S2.** Comparison of absolute Pearson correlations coefficients between datasets with imputation and without imputation using different interpolations methods (Gompertz vs. linear vs. random)


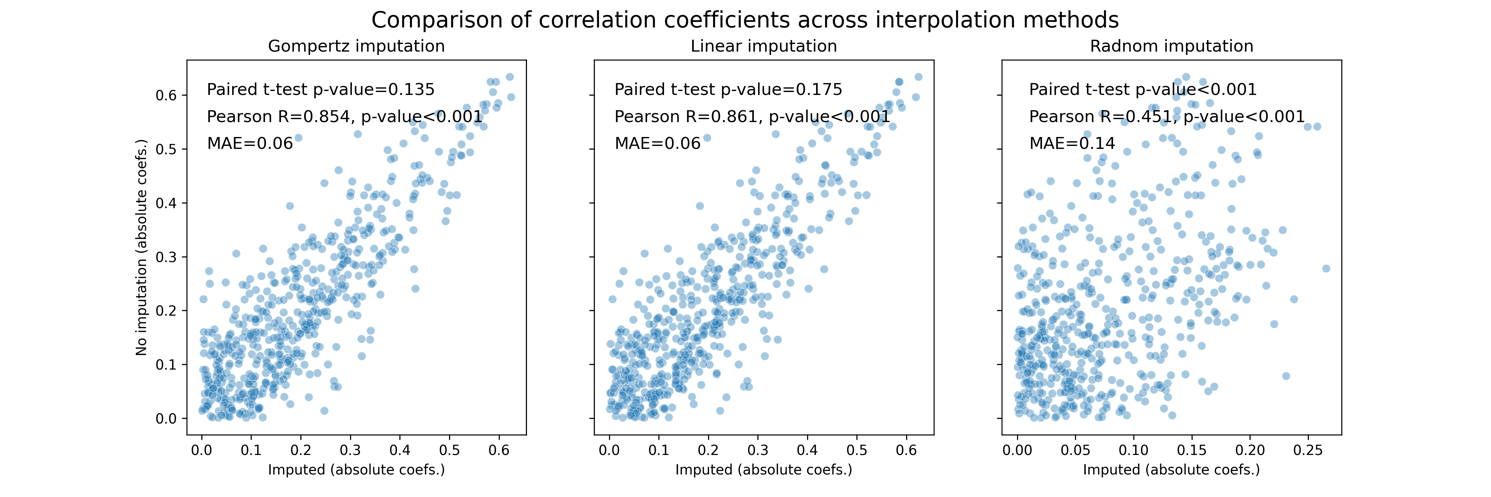


**Figure S3.** Comparison of absolute Pearson correlations coefficients between datasets with imputation using different interpolations methods (Gompertz vs. linear and Gompertz vs. random)


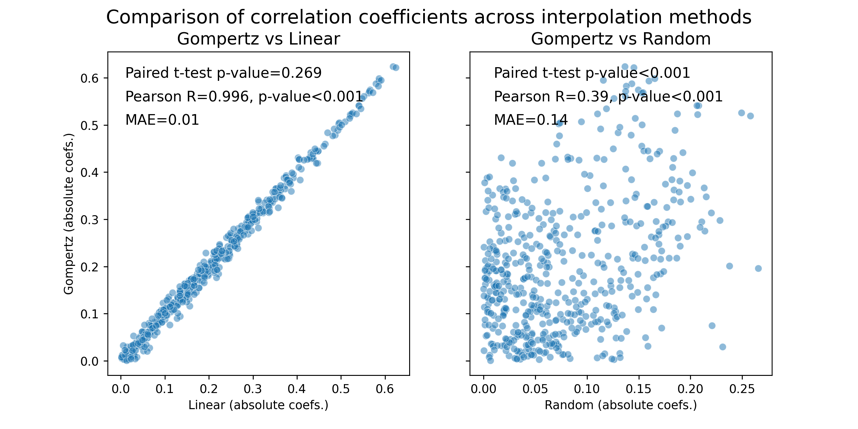


**Table S2.** Spearman and Pearson correlations between absolute NTB composite scores and physiological features (only significant results are included with both Pearson and Spearman p-values <0.05 after the FDR correction)

| Outcome | Feature | Rho (Spearman) | Rho  p-value | Rho p-value adjusted | R (Pearson) | R  p-value | R p-value adjusted |
| --- | --- | --- | --- | --- | --- | --- | --- |
| NTB_Global | HR_Kurt | -0.37 | 2.5E-04 | 1.9E-03 | -0.46 | 2.4E-06 | 3.9E-05 |
| NTB_Global | HR_Skew | -0.35 | 4.4E-04 | 2.9E-03 | -0.43 | 1.4E-05 | 1.6E-04 |
| NTB_Global | IBI_HRV_CSI | -0.49 | 3.9E-07 | 1.1E-05 | -0.52 | 5.9E-08 | 1.5E-06 |
| NTB_Global | IBI_HRV_CSI_Mod | -0.34 | 7.3E-04 | 4.4E-03 | -0.41 | 3.9E-05 | 3.8E-04 |
| NTB_Global | IBI_HRV_HF | 0.39 | 6.8E-05 | 6.4E-04 | 0.54 | 1.2E-08 | 4.4E-07 |
| NTB_Global | IBI_HRV_HFn | 0.44 | 9.4E-06 | 1.4E-04 | 0.46 | 3.1E-06 | 4.9E-05 |
| NTB_Global | IBI_HRV_IALS | 0.30 | 3.4E-03 | 1.5E-02 | 0.28 | 6.6E-03 | 2.4E-02 |
| NTB_Global | IBI_HRV_LFHF | -0.41 | 3.1E-05 | 3.5E-04 | -0.48 | 8.1E-07 | 1.4E-05 |
| NTB_Global | IBI_HRV_LFn | -0.34 | 5.8E-04 | 3.6E-03 | -0.36 | 2.6E-04 | 1.9E-03 |
| NTB_Global | IBI_HRV_LnHF | 0.41 | 3.7E-05 | 3.8E-04 | 0.52 | 4.9E-08 | 1.3E-06 |
| NTB_Global | IBI_HRV_NNkurt | -0.27 | 8.4E-03 | 3.2E-02 | -0.38 | 1.2E-04 | 1.0E-03 |
| NTB_Global | IBI_HRV_PSS | 0.29 | 4.4E-03 | 1.8E-02 | 0.26 | 9.6E-03 | 3.2E-02 |
| NTB_Global | IBI_HRV_SD1SD2 | 0.48 | 5.9E-07 | 1.6E-05 | 0.53 | 3.8E-08 | 1.2E-06 |
| NTB_Global | IBI_HRV_VHF | 0.46 | 3.1E-06 | 5.3E-05 | 0.44 | 6.7E-06 | 9.1E-05 |
| NTB_Global | IBI_HRV_VLF | -0.30 | 3.3E-03 | 1.5E-02 | -0.31 | 1.8E-03 | 8.8E-03 |
| NTB_Global | PPG_HRV_CSI | -0.43 | 1.3E-05 | 1.8E-04 | -0.45 | 5.6E-06 | 7.9E-05 |
| NTB_Global | PPG_HRV_CSI_Mod | -0.33 | 1.2E-03 | 6.7E-03 | -0.39 | 9.4E-05 | 8.3E-04 |
| NTB_Global | PPG_HRV_HF | 0.43 | 1.3E-05 | 1.8E-04 | 0.54 | 1.3E-08 | 4.4E-07 |
| NTB_Global | PPG_HRV_HFn | 0.42 | 2.5E-05 | 3.1E-04 | 0.43 | 1.2E-05 | 1.5E-04 |
| NTB_Global | PPG_HRV_LFHF | -0.37 | 2.2E-04 | 1.7E-03 | -0.43 | 1.3E-05 | 1.5E-04 |
| NTB_Global | PPG_HRV_LFn | -0.32 | 1.7E-03 | 9.1E-03 | -0.35 | 4.4E-04 | 2.9E-03 |
| NTB_Global | PPG_HRV_LnHF | 0.43 | 1.0E-05 | 1.5E-04 | 0.52 | 4.4E-08 | 1.2E-06 |
| NTB_Global | PPG_HRV_NNskew | 0.25 | 1.3E-02 | 4.5E-02 | 0.32 | 1.4E-03 | 7.0E-03 |
| NTB_Global | PPG_HRV_SD1SD2 | 0.42 | 2.3E-05 | 2.9E-04 | 0.44 | 5.7E-06 | 7.9E-05 |
| NTB_Global | PPG_HRV_VHF | 0.39 | 9.0E-05 | 7.8E-04 | 0.34 | 6.6E-04 | 4.0E-03 |
| NTB_Global | PPG_HRV_VLF | -0.26 | 1.1E-02 | 3.7E-02 | -0.30 | 2.8E-03 | 1.2E-02 |
| NTB_Global | PPG_HR_PSD_VLF | -0.26 | 9.9E-03 | 3.6E-02 | -0.34 | 8.4E-04 | 4.6E-03 |
| NTB_ExecFunc | EDAsymp | 0.45 | 4.2E-06 | 6.8E-05 | 0.48 | 8.8E-07 | 1.4E-05 |
| NTB_ExecFunc | EDAsymp2N | 0.38 | 1.1E-04 | 8.9E-04 | 0.51 | 1.3E-07 | 3.1E-06 |
| NTB_ExecFunc | HR_Kurt | -0.37 | 2.0E-04 | 1.5E-03 | -0.43 | 1.1E-05 | 1.4E-04 |
| NTB_ExecFunc | HR_Max | -0.27 | 6.9E-03 | 2.8E-02 | -0.28 | 6.6E-03 | 2.4E-02 |
| NTB_ExecFunc | HR_Skew | -0.46 | 2.1E-06 | 3.9E-05 | -0.48 | 6.0E-07 | 1.1E-05 |
| NTB_ExecFunc | HR_Std | -0.34 | 5.8E-04 | 3.6E-03 | -0.34 | 7.2E-04 | 4.2E-03 |
| NTB_ExecFunc | IBI_HRV_CSI | -0.59 | 1.7E-10 | 2.9E-08 | -0.59 | 1.9E-10 | 2.0E-08 |
| NTB_ExecFunc | IBI_HRV_CSI_Mod | -0.44 | 8.3E-06 | 1.3E-04 | -0.45 | 5.4E-06 | 7.9E-05 |
| NTB_ExecFunc | IBI_HRV_HF | 0.52 | 5.0E-08 | 2.2E-06 | 0.57 | 9.1E-10 | 6.0E-08 |
| NTB_ExecFunc | IBI_HRV_HFn | 0.62 | 1.7E-11 | 9.3E-09 | 0.62 | 1.1E-11 | 3.7E-09 |
| NTB_ExecFunc | IBI_HRV_LFHF | -0.60 | 7.7E-11 | 2.0E-08 | -0.62 | 1.4E-11 | 3.7E-09 |
| NTB_ExecFunc | IBI_HRV_LFn | -0.49 | 3.3E-07 | 1.0E-05 | -0.52 | 4.4E-08 | 1.2E-06 |
| NTB_ExecFunc | IBI_HRV_LnHF | 0.55 | 5.3E-09 | 2.8E-07 | 0.60 | 1.2E-10 | 2.0E-08 |
| NTB_ExecFunc | IBI_HRV_NNskew | 0.28 | 6.0E-03 | 2.5E-02 | 0.31 | 2.4E-03 | 1.1E-02 |
| NTB_ExecFunc | IBI_HRV_SD1SD2 | 0.57 | 1.1E-09 | 9.9E-08 | 0.59 | 3.2E-10 | 2.8E-08 |
| NTB_ExecFunc | IBI_HRV_VHF | 0.31 | 1.8E-03 | 9.4E-03 | 0.31 | 2.4E-03 | 1.1E-02 |
| NTB_ExecFunc | IBI_HRV_VLF | -0.31 | 2.2E-03 | 1.0E-02 | -0.34 | 7.2E-04 | 4.2E-03 |
| NTB_ExecFunc | PPG_HRV_CSI | -0.58 | 4.2E-10 | 4.5E-08 | -0.57 | 1.6E-09 | 7.5E-08 |
| NTB_ExecFunc | PPG_HRV_CSI_Mod | -0.44 | 8.4E-06 | 1.3E-04 | -0.43 | 1.3E-05 | 1.5E-04 |
| NTB_ExecFunc | PPG_HRV_HF | 0.51 | 1.1E-07 | 4.3E-06 | 0.56 | 2.8E-09 | 1.3E-07 |
| NTB_ExecFunc | PPG_HRV_HFn | 0.59 | 2.2E-10 | 3.0E-08 | 0.60 | 1.6E-10 | 2.0E-08 |
| NTB_ExecFunc | PPG_HRV_LF | -0.34 | 8.0E-04 | 4.7E-03 | -0.27 | 8.1E-03 | 2.8E-02 |
| NTB_ExecFunc | PPG_HRV_LFHF | -0.56 | 4.3E-09 | 2.5E-07 | -0.58 | 4.8E-10 | 3.6E-08 |
| NTB_ExecFunc | PPG_HRV_LFn | -0.49 | 3.1E-07 | 1.0E-05 | -0.53 | 2.0E-08 | 6.7E-07 |
| NTB_ExecFunc | PPG_HRV_LnHF | 0.52 | 6.8E-08 | 2.8E-06 | 0.56 | 4.0E-09 | 1.6E-07 |
| NTB_ExecFunc | PPG_HRV_PIP | 0.30 | 3.4E-03 | 1.5E-02 | 0.29 | 3.7E-03 | 1.5E-02 |
| NTB_ExecFunc | PPG_HRV_PSS | 0.35 | 4.6E-04 | 2.9E-03 | 0.35 | 5.2E-04 | 3.4E-03 |
| NTB_ExecFunc | PPG_HRV_SD1SD2 | 0.57 | 1.4E-09 | 1.0E-07 | 0.57 | 1.2E-09 | 6.9E-08 |
| NTB_ExecFunc | PPG_HRV_VHF | 0.30 | 3.4E-03 | 1.5E-02 | 0.28 | 5.5E-03 | 2.1E-02 |
| NTB_ExecFunc | PPG_HRV_VLF | -0.31 | 1.9E-03 | 9.7E-03 | -0.36 | 3.1E-04 | 2.2E-03 |
| NTB_ExecFunc | PPG_HR_PSD_LF | -0.36 | 2.6E-04 | 1.9E-03 | -0.33 | 1.1E-03 | 6.1E-03 |
| NTB_ExecFunc | PPG_HR_PSD_VLF | -0.47 | 1.3E-06 | 3.0E-05 | -0.44 | 7.8E-06 | 1.0E-04 |
| NTB_ProcSpeed | HR_Mean | 0.38 | 1.5E-04 | 1.2E-03 | 0.37 | 2.5E-04 | 1.8E-03 |
| NTB_ProcSpeed | HR_Min | 0.40 | 5.3E-05 | 5.0E-04 | 0.39 | 9.4E-05 | 8.3E-04 |
| NTB_ProcSpeed | IBI_HRV_BSI | 0.36 | 2.8E-04 | 2.0E-03 | 0.34 | 7.8E-04 | 4.5E-03 |
| NTB_ProcSpeed | IBI_HRV_CSI | -0.31 | 2.0E-03 | 1.0E-02 | -0.36 | 2.8E-04 | 2.0E-03 |
| NTB_ProcSpeed | IBI_HRV_CSI_Mod | -0.41 | 3.1E-05 | 3.5E-04 | -0.38 | 1.3E-04 | 1.0E-03 |
| NTB_ProcSpeed | IBI_HRV_CVI | -0.29 | 4.2E-03 | 1.8E-02 | -0.26 | 1.0E-02 | 3.3E-02 |
| NTB_ProcSpeed | IBI_HRV_HFn | 0.30 | 2.7E-03 | 1.3E-02 | 0.30 | 3.4E-03 | 1.5E-02 |
| NTB_ProcSpeed | IBI_HRV_HTI | -0.36 | 2.9E-04 | 2.0E-03 | -0.26 | 9.6E-03 | 3.2E-02 |
| NTB_ProcSpeed | IBI_HRV_IALS | 0.47 | 1.1E-06 | 2.7E-05 | 0.50 | 1.8E-07 | 3.8E-06 |
| NTB_ProcSpeed | IBI_HRV_IQRNN | -0.39 | 7.8E-05 | 7.0E-04 | -0.27 | 9.0E-03 | 3.0E-02 |
| NTB_ProcSpeed | IBI_HRV_LF | -0.56 | 2.0E-09 | 1.3E-07 | -0.43 | 1.2E-05 | 1.5E-04 |
| NTB_ProcSpeed | IBI_HRV_LFHF | -0.45 | 3.9E-06 | 6.5E-05 | -0.49 | 4.3E-07 | 7.9E-06 |
| NTB_ProcSpeed | IBI_HRV_LFn | -0.53 | 3.7E-08 | 1.8E-06 | -0.49 | 3.6E-07 | 6.7E-06 |
| NTB_ProcSpeed | IBI_HRV_PIP | 0.46 | 1.9E-06 | 3.8E-05 | 0.50 | 1.6E-07 | 3.5E-06 |
| NTB_ProcSpeed | IBI_HRV_PSS | 0.47 | 1.4E-06 | 3.0E-05 | 0.57 | 1.5E-09 | 7.5E-08 |
| NTB_ProcSpeed | IBI_HRV_SD1SD2 | 0.30 | 2.9E-03 | 1.4E-02 | 0.29 | 4.4E-03 | 1.7E-02 |
| NTB_ProcSpeed | IBI_HRV_SD2 | -0.41 | 2.9E-05 | 3.4E-04 | -0.34 | 8.3E-04 | 4.6E-03 |
| NTB_ProcSpeed | IBI_HRV_SDNN | -0.39 | 8.4E-05 | 7.5E-04 | -0.31 | 2.5E-03 | 1.1E-02 |
| NTB_ProcSpeed | IBI_HRV_TINN | -0.40 | 4.4E-05 | 4.4E-04 | -0.32 | 1.6E-03 | 8.2E-03 |
| NTB_ProcSpeed | PPG_BSI | 0.40 | 4.4E-05 | 4.4E-04 | 0.43 | 1.4E-05 | 1.6E-04 |
| NTB_ProcSpeed | PPG_BVP_HF | 0.46 | 2.9E-06 | 5.1E-05 | 0.37 | 2.5E-04 | 1.8E-03 |
| NTB_ProcSpeed | PPG_BVP_MF | 0.37 | 1.8E-04 | 1.4E-03 | 0.34 | 8.2E-04 | 4.6E-03 |
| NTB_ProcSpeed | PPG_HRV_AI | 0.26 | 9.8E-03 | 3.6E-02 | 0.28 | 6.5E-03 | 2.4E-02 |
| NTB_ProcSpeed | PPG_HRV_CSI | -0.31 | 2.1E-03 | 1.0E-02 | -0.33 | 1.1E-03 | 6.0E-03 |
| NTB_ProcSpeed | PPG_HRV_CSI_Mod | -0.42 | 1.8E-05 | 2.4E-04 | -0.37 | 1.7E-04 | 1.3E-03 |
| NTB_ProcSpeed | PPG_HRV_CVI | -0.42 | 1.9E-05 | 2.5E-04 | -0.39 | 7.4E-05 | 6.9E-04 |
| NTB_ProcSpeed | PPG_HRV_GI | 0.30 | 3.5E-03 | 1.5E-02 | 0.31 | 1.8E-03 | 8.8E-03 |
| NTB_ProcSpeed | PPG_HRV_HFn | 0.30 | 3.2E-03 | 1.5E-02 | 0.29 | 4.7E-03 | 1.8E-02 |
| NTB_ProcSpeed | PPG_HRV_HTI | -0.40 | 4.7E-05 | 4.5E-04 | -0.29 | 4.7E-03 | 1.8E-02 |
| NTB_ProcSpeed | PPG_HRV_IALS | 0.47 | 1.1E-06 | 2.7E-05 | 0.49 | 3.0E-07 | 5.8E-06 |
| NTB_ProcSpeed | PPG_HRV_IQRNN | -0.40 | 4.4E-05 | 4.4E-04 | -0.27 | 7.5E-03 | 2.6E-02 |
| NTB_ProcSpeed | PPG_HRV_LF | -0.46 | 2.3E-06 | 4.2E-05 | -0.34 | 6.8E-04 | 4.0E-03 |
| NTB_ProcSpeed | PPG_HRV_LFHF | -0.36 | 3.8E-04 | 2.5E-03 | -0.35 | 4.1E-04 | 2.7E-03 |
| NTB_ProcSpeed | PPG_HRV_LFn | -0.41 | 3.0E-05 | 3.5E-04 | -0.40 | 6.5E-05 | 6.1E-04 |
| NTB_ProcSpeed | PPG_HRV_PAS | 0.47 | 1.4E-06 | 3.1E-05 | 0.43 | 1.4E-05 | 1.6E-04 |
| NTB_ProcSpeed | PPG_HRV_PIP | 0.47 | 1.1E-06 | 2.7E-05 | 0.50 | 2.1E-07 | 4.2E-06 |
| NTB_ProcSpeed | PPG_HRV_PSS | 0.50 | 1.8E-07 | 6.2E-06 | 0.51 | 8.3E-08 | 2.0E-06 |
| NTB_ProcSpeed | PPG_HRV_Prc20NN | -0.34 | 6.0E-04 | 3.7E-03 | -0.37 | 2.5E-04 | 1.8E-03 |
| NTB_ProcSpeed | PPG_HRV_Prc80NN | -0.38 | 1.1E-04 | 9.0E-04 | -0.39 | 8.6E-05 | 7.8E-04 |
| NTB_ProcSpeed | PPG_HRV_RMSSD | -0.42 | 2.5E-05 | 3.1E-04 | -0.28 | 5.8E-03 | 2.1E-02 |
| NTB_ProcSpeed | PPG_HRV_SD1SD2 | 0.29 | 3.9E-03 | 1.7E-02 | 0.29 | 4.8E-03 | 1.8E-02 |
| NTB_ProcSpeed | PPG_HRV_SDNN | -0.47 | 1.5E-06 | 3.2E-05 | -0.38 | 1.1E-04 | 9.5E-04 |
| NTB_ProcSpeed | PPG_HRV_SI | 0.28 | 4.9E-03 | 2.0E-02 | 0.25 | 1.5E-02 | 4.6E-02 |
| NTB_ProcSpeed | PPG_HR_mean | 0.36 | 3.5E-04 | 2.3E-03 | 0.36 | 3.4E-04 | 2.3E-03 |
| NTB_MemoryImm | HR_Kurt | -0.26 | 1.2E-02 | 4.2E-02 | -0.34 | 6.3E-04 | 3.9E-03 |
| NTB_MemoryImm | IBI_HRV_CSI | -0.34 | 6.9E-04 | 4.1E-03 | -0.37 | 2.4E-04 | 1.8E-03 |
| NTB_MemoryImm | IBI_HRV_HF | 0.27 | 7.0E-03 | 2.8E-02 | 0.40 | 5.8E-05 | 5.6E-04 |
| NTB_MemoryImm | IBI_HRV_HFn | 0.27 | 7.2E-03 | 2.8E-02 | 0.28 | 6.7E-03 | 2.4E-02 |
| NTB_MemoryImm | IBI_HRV_LnHF | 0.28 | 6.1E-03 | 2.5E-02 | 0.36 | 3.4E-04 | 2.3E-03 |
| NTB_MemoryImm | IBI_HRV_SD1SD2 | 0.34 | 6.5E-04 | 3.9E-03 | 0.37 | 1.9E-04 | 1.5E-03 |
| NTB_MemoryImm | IBI_HRV_VHF | 0.39 | 9.4E-05 | 8.0E-04 | 0.43 | 1.5E-05 | 1.6E-04 |
| NTB_MemoryImm | PPG_BVP_MF | -0.25 | 1.3E-02 | 4.3E-02 | -0.25 | 1.5E-02 | 4.7E-02 |
| NTB_MemoryImm | PPG_HRV_CSI | -0.26 | 9.7E-03 | 3.5E-02 | -0.28 | 5.1E-03 | 2.0E-02 |
| NTB_MemoryImm | PPG_HRV_HF | 0.31 | 1.8E-03 | 9.4E-03 | 0.41 | 3.3E-05 | 3.3E-04 |
| NTB_MemoryImm | PPG_HRV_HFn | 0.26 | 1.1E-02 | 3.9E-02 | 0.26 | 9.4E-03 | 3.2E-02 |
| NTB_MemoryImm | PPG_HRV_LnHF | 0.31 | 1.8E-03 | 9.3E-03 | 0.38 | 1.1E-04 | 9.5E-04 |
| NTB_MemoryImm | PPG_HRV_SD1SD2 | 0.25 | 1.2E-02 | 4.2E-02 | 0.27 | 6.8E-03 | 2.4E-02 |
| NTB_MemoryImm | PPG_HRV_VHF | 0.32 | 1.3E-03 | 7.3E-03 | 0.31 | 2.1E-03 | 9.6E-03 |
| NTB_MemoryImm | PPG_HR_PSD_HF | 0.25 | 1.3E-02 | 4.4E-02 | 0.25 | 1.2E-02 | 3.9E-02 |
| NTB_MemoryDel | HR_Kurt | -0.35 | 4.6E-04 | 3.0E-03 | -0.35 | 5.3E-04 | 3.4E-03 |
| NTB_MemoryDel | HR_Skew | -0.27 | 7.1E-03 | 2.8E-02 | -0.35 | 5.7E-04 | 3.6E-03 |
| NTB_MemoryDel | IBI_HRV_CSI | -0.32 | 1.4E-03 | 7.7E-03 | -0.28 | 5.4E-03 | 2.0E-02 |
| NTB_MemoryDel | IBI_HRV_CVSD | 0.27 | 8.0E-03 | 3.0E-02 | 0.38 | 1.5E-04 | 1.2E-03 |
| NTB_MemoryDel | IBI_HRV_HF | 0.32 | 1.3E-03 | 7.1E-03 | 0.42 | 2.1E-05 | 2.2E-04 |
| NTB_MemoryDel | IBI_HRV_LnHF | 0.32 | 1.4E-03 | 7.7E-03 | 0.36 | 3.2E-04 | 2.2E-03 |
| NTB_MemoryDel | IBI_HRV_NNkurt | -0.27 | 8.0E-03 | 3.0E-02 | -0.30 | 3.1E-03 | 1.3E-02 |
| NTB_MemoryDel | IBI_HRV_SD1SD2 | 0.33 | 1.2E-03 | 6.8E-03 | 0.32 | 1.2E-03 | 6.5E-03 |
| NTB_MemoryDel | IBI_HRV_VHF | 0.41 | 3.2E-05 | 3.5E-04 | 0.32 | 1.7E-03 | 8.3E-03 |
| NTB_MemoryDel | PPG_BVP_MF | -0.30 | 3.0E-03 | 1.4E-02 | -0.32 | 1.3E-03 | 6.9E-03 |
| NTB_MemoryDel | PPG_HRV_HF | 0.36 | 3.5E-04 | 2.3E-03 | 0.42 | 2.2E-05 | 2.2E-04 |
| NTB_MemoryDel | PPG_HRV_LnHF | 0.36 | 3.0E-04 | 2.1E-03 | 0.38 | 1.2E-04 | 9.7E-04 |
| NTB_MemoryDel | PPG_HR_PSD_HF | 0.39 | 7.4E-05 | 6.8E-04 | 0.34 | 6.4E-04 | 3.9E-03 |
| NTB_MemoryDel | Temp_IQR | 0.39 | 1.1E-04 | 8.9E-04 | 0.43 | 1.5E-05 | 1.6E-04 |
| NTB_MemoryDel | Temp_Kurt | -0.33 | 1.2E-03 | 6.7E-03 | -0.31 | 2.4E-03 | 1.1E-02 |
| NTB_MemoryDel | Temp_Std | 0.41 | 3.3E-05 | 3.5E-04 | 0.45 | 4.3E-06 | 6.6E-05 |

**Table S3.** Spearman and Pearson correlations between intra-individual changes in NTB composite scores and intra-individual changes in physiological features (centered dataset, only significant results are included with both Pearson and Spearman p-values <0.05 after the FDR correction)

| Outcome | Feature | Rho (Spearman) | Rho  p-value | Rho  p-value adjusted | R (Pearson) | R p-value | R p-value adjusted |
| --- | --- | --- | --- | --- | --- | --- | --- |
| NTB_ExecFunc | HR_Max | 0.38 | 1.2E-04 | 1.2E-02 | 0.60 | 7.4E-11 | 6.5E-09 |
| NTB_ExecFunc | HR_Mean | 0.38 | 1.6E-04 | 1.3E-02 | 0.66 | 3.2E-13 | 7.1E-11 |
| NTB_ExecFunc | HR_Min | 0.39 | 7.2E-05 | 1.2E-02 | 0.70 | 3.6E-15 | 1.9E-12 |
| NTB_ExecFunc | IBI_HRV_BSI | 0.32 | 1.6E-03 | 4.4E-02 | 0.61 | 4.6E-11 | 4.8E-09 |
| NTB_ExecFunc | IBI_HRV_CSI_Mod | -0.36 | 3.2E-04 | 1.7E-02 | -0.44 | 5.9E-06 | 1.0E-04 |
| NTB_ExecFunc | IBI_HRV_HTI | -0.35 | 5.0E-04 | 1.9E-02 | -0.44 | 5.6E-06 | 9.9E-05 |
| NTB_ExecFunc | IBI_HRV_IALS | 0.35 | 4.0E-04 | 1.8E-02 | 0.58 | 4.3E-10 | 2.1E-08 |
| NTB_ExecFunc | IBI_HRV_PAS | 0.38 | 1.1E-04 | 1.2E-02 | 0.59 | 2.1E-10 | 1.2E-08 |
| NTB_ExecFunc | IBI_HRV_PIP | 0.37 | 2.6E-04 | 1.6E-02 | 0.59 | 1.7E-10 | 1.1E-08 |
| NTB_ExecFunc | IBI_HRV_SD2 | -0.35 | 4.3E-04 | 1.8E-02 | -0.50 | 2.1E-07 | 6.3E-06 |
| NTB_ExecFunc | IBI_HRV_VHF | 0.43 | 1.1E-05 | 4.5E-03 | 0.41 | 3.7E-05 | 5.4E-04 |
| NTB_ExecFunc | PPG_BSI | 0.34 | 7.4E-04 | 2.5E-02 | 0.54 | 9.5E-09 | 3.9E-07 |
| NTB_ExecFunc | PPG_HRV_CVI | -0.37 | 1.7E-04 | 1.3E-02 | -0.52 | 6.7E-08 | 2.4E-06 |
| NTB_ExecFunc | PPG_HRV_HTI | -0.34 | 7.5E-04 | 2.5E-02 | -0.46 | 1.9E-06 | 4.6E-05 |
| NTB_ExecFunc | PPG_HRV_Prc20NN | -0.35 | 4.4E-04 | 1.8E-02 | -0.59 | 2.9E-10 | 1.5E-08 |
| NTB_ExecFunc | PPG_HRV_Prc80NN | -0.42 | 1.7E-05 | 4.5E-03 | -0.62 | 1.8E-11 | 2.4E-09 |
| NTB_ExecFunc | PPG_HRV_SDNN | -0.32 | 1.6E-03 | 4.4E-02 | -0.41 | 2.8E-05 | 4.3E-04 |
| NTB_ExecFunc | PPG_HR_mean | 0.36 | 2.8E-04 | 1.6E-02 | 0.66 | 4.0E-13 | 7.1E-11 |
| NTB_ProcSpeed | IBI_HRV_SD1SD2 | 0.33 | 9.4E-04 | 2.9E-02 | 0.47 | 1.2E-06 | 3.1E-05 |

**Table S4.** Linear mixed-effects regression results (only significant results are included with adjusted p-values <0.05 after the FDR correction)

| Outcome | Feature | Coef. | t-value | R2 | p-value | p-value adjusted |
| --- | --- | --- | --- | --- | --- | --- |
| NTB_Global | IBI_HRV_CVI | -0.486 | -3.64 | 0.74 | 2.7E-04 | 6.5E-03 |
| NTB_Global | IBI_HRV_PIP | 1.991 | 3.05 | 0.55 | 2.3E-03 | 3.5E-02 |
| NTB_Global | IBI_HRV_RMSSD | -0.020 | -3.03 | 0.89 | 2.4E-03 | 3.5E-02 |
| NTB_Global | IBI_HRV_VHF | 73.209 | 3.60 | 0.50 | 3.2E-04 | 7.5E-03 |
| NTB_Global | PPG_HRV_CVI | -0.443 | -3.96 | 0.66 | 7.4E-05 | 1.9E-03 |
| NTB_Global | PPG_HRV_PI | -0.050 | -2.94 | 0.94 | 3.2E-03 | 4.3E-02 |
| NTB_Global | PPG_HRV_Prc20NN | -0.001 | -3.25 | 0.57 | 1.1E-03 | 1.9E-02 |
| NTB_Global | PPG_HRV_Prc80NN | -0.001 | -3.49 | 0.59 | 4.9E-04 | 1.0E-02 |
| NTB_Global | PPG_HRV_VHF | 65.269 | 3.38 | 0.49 | 7.3E-04 | 1.3E-02 |
| NTB_ExecFunc | HR_Max | 0.030 | 6.27 | 0.37 | 3.7E-10 | 3.9E-08 |
| NTB_ExecFunc | HR_Mean | 0.031 | 5.63 | 0.37 | 1.8E-08 | 8.1E-07 |
| NTB_ExecFunc | HR_Min | 0.033 | 6.44 | 0.44 | 1.2E-10 | 2.2E-08 |
| NTB_ExecFunc | IBI_HRV_C1d | -4.134 | -3.42 | 0.77 | 6.2E-04 | 1.2E-02 |
| NTB_ExecFunc | IBI_HRV_CSI | -0.249 | -2.92 | 0.62 | 3.6E-03 | 4.5E-02 |
| NTB_ExecFunc | IBI_HRV_CVI | -0.665 | -5.75 | 0.97 | 9.0E-09 | 5.5E-07 |
| NTB_ExecFunc | IBI_HRV_HTI | -0.114 | -3.20 | 0.52 | 1.4E-03 | 2.2E-02 |
| NTB_ExecFunc | IBI_HRV_IALS | 3.300 | 6.31 | 0.47 | 2.9E-10 | 3.8E-08 |
| NTB_ExecFunc | IBI_HRV_PAS | 1.407 | 3.17 | 0.37 | 1.6E-03 | 2.4E-02 |
| NTB_ExecFunc | IBI_HRV_PI | -0.050 | -3.31 | 0.95 | 9.4E-04 | 1.7E-02 |
| NTB_ExecFunc | IBI_HRV_PIP | 3.661 | 6.52 | 0.47 | 6.9E-11 | 1.8E-08 |
| NTB_ExecFunc | IBI_HRV_PSS | 3.732 | 5.18 | 0.48 | 2.2E-07 | 7.7E-06 |
| NTB_ExecFunc | IBI_HRV_VHF | 106.836 | 5.42 | 0.52 | 6.0E-08 | 2.5E-06 |
| NTB_ExecFunc | PPG_HRV_CVI | -0.659 | -6.79 | 0.97 | 1.1E-11 | 6.1E-09 |
| NTB_ExecFunc | PPG_HRV_HTI | -0.102 | -3.04 | 0.52 | 2.4E-03 | 3.5E-02 |
| NTB_ExecFunc | PPG_HRV_IALS | 2.864 | 5.73 | 0.45 | 1.0E-08 | 5.5E-07 |
| NTB_ExecFunc | PPG_HRV_MadNN | -0.010 | -2.95 | 0.89 | 3.2E-03 | 4.3E-02 |
| NTB_ExecFunc | PPG_HRV_PIP | 4.155 | 5.40 | 0.47 | 6.7E-08 | 2.5E-06 |
| NTB_ExecFunc | PPG_HRV_PSS | 4.225 | 4.53 | 0.48 | 6.0E-06 | 1.7E-04 |
| NTB_ExecFunc | PPG_HRV_Prc20NN | -0.002 | -5.96 | 0.64 | 2.6E-09 | 1.9E-07 |
| NTB_ExecFunc | PPG_HRV_Prc80NN | -0.002 | -6.13 | 0.65 | 8.6E-10 | 7.6E-08 |
| NTB_ExecFunc | PPG_HRV_SDNN | -0.009 | -3.00 | 0.56 | 2.7E-03 | 3.7E-02 |
| NTB_ExecFunc | PPG_HRV_VHF | 100.174 | 5.71 | 0.51 | 1.1E-08 | 5.5E-07 |
| NTB_ExecFunc | PPG_HR_mean | 0.031 | 5.72 | 0.37 | 1.1E-08 | 5.5E-07 |
| NTB_ProcSpeed | IBI_HRV_C2d | -3.641 | -2.93 | 0.93 | 3.4E-03 | 4.4E-02 |
| NTB_ProcSpeed | IBI_HRV_CVSD | 12.137 | 4.76 | 0.47 | 1.9E-06 | 5.7E-05 |
| NTB_ProcSpeed | IBI_HRV_HF | 25.222 | 4.93 | 0.48 | 8.4E-07 | 2.8E-05 |
| NTB_ProcSpeed | IBI_HRV_HFn | 2.099 | 3.49 | 0.49 | 4.9E-04 | 1.0E-02 |
| NTB_ProcSpeed | IBI_HRV_PI | 0.080 | 3.24 | 0.83 | 1.2E-03 | 2.0E-02 |
| NTB_ProcSpeed | IBI_HRV_SD1SD2 | 1.587 | 4.89 | 0.50 | 1.0E-06 | 3.2E-05 |
| NTB_ProcSpeed | IBI_HRV_pNN20 | 0.017 | 3.47 | 0.41 | 5.1E-04 | 1.0E-02 |
| NTB_ProcSpeed | PPG_HRV_HFn | 1.928 | 3.52 | 0.49 | 4.4E-04 | 9.7E-03 |
| NTB_ProcSpeed | PPG_HRV_SD1SD2 | 1.733 | 3.97 | 0.50 | 7.3E-05 | 1.9E-03 |

**Table S5.** Features used in the best models predicting NTB composite scores (as reported in the Table 2 of the main paper)

| Outcomes | NTB_Global | NTB_ExecFunc | NTB_ProcSpeed | NTB_MemoryImm | NTB_MemoryDel |
| --- | --- | --- | --- | --- | --- |
| Features used in subject-based cross-validation | HR_Skew IBI_HRV_CSI_Mod IBI_HRV_LFHF  IBI_HRV_HF  IBI_HRV_HFn  PPG_HRV_VHF  HR_Kurt  PPG_HRV_LFHF  IBI_HRV_VHF  Age  Gender | PPG_HRV_PSS  EDAsymp2N  HR_Skew  IBI_HRV_CSI_Mod  EDAsymp  IBI_HRV_LFHF  IBI_HRV_HF  IBI_HRV_HFn  HR_Kurt  HR_Std  PPG_HR_PSD_VLF  Age  Gender | IBI_HRV_IALS  HR_Min  IBI_HRV_CSI_Mod  IBI_HRV_LFHF  PPG_BSI  IBI_HRV_SD2  IBI_HRV_LF  PPG_HRV_LFHF  PPG_BVP_MF  IBI_HRV_PSS  PPG_HRV_PAS  PPG_BVP_HF  Age  Gender | IBI_HRV_VHF IBI_HRV_SD1SD2  Age  Gender | PPG_HR_PSD_HF  PPG_HRV_HF  HR_Kurt  Temp_IQR  Age  Gender |
| Features used in interval-based cross-validation | PPG_HRV_HFn  IBI_HRV_LFHF  IBI_HRV_HF  IBI_HRV_HFn  IBI_HRV_VHF  Age  Gender | HR_Skew IBI_HRV_CSI_Mod  EDAsymp  IBI_HRV_LFHF  IBI_HRV_HF  IBI_HRV_HFn  PPG_HR_PSD_VLF  Age  Gender | IBI_HRV_IALS  HR_Min  IBI_HRV_LFHF  PPG_BSI  IBI_HRV_LF  IBI_HRV_PSS  PPG_HRV_PAS  PPG_HRV_IALS  PPG_HRV_CVI  Age  Gender | IBI_HRV_VHF  Age  Gender | Temp_Std  Age  Gender |

**Figure S4.** NTB composite scores: changes from baseline to post-intervention


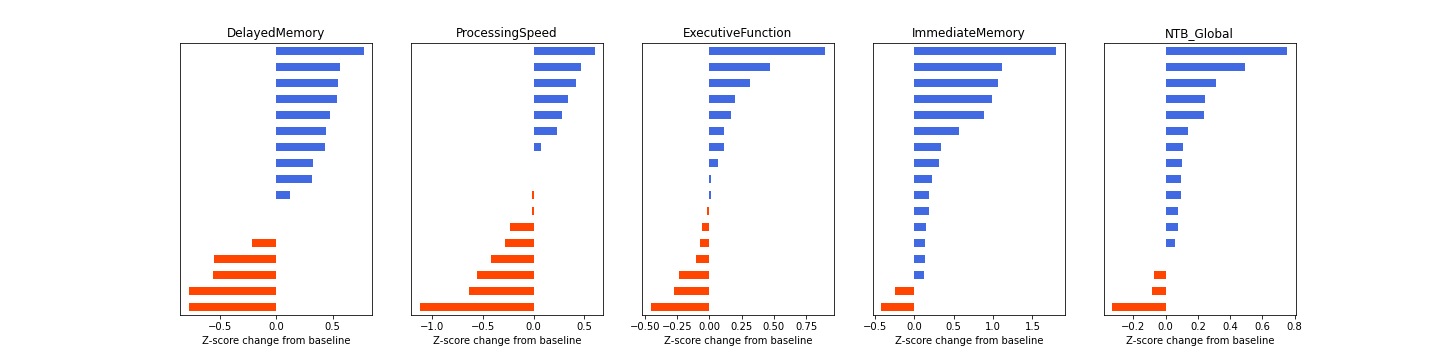


Bars represent changes in z-score from baseline for each participant (blue – positive change, improvement, red – negative change, decline). Delayed memory, processing speed and executive function showed mixed results with changes in different directions (both improvement and decline), while immediate memory and global cognition were mostly changed in one direction (improvement).

**Table S6.** Mean and standard deviation of NTB composite scores at the baseline and post-intervention assessments (N=17)

| NTB Composites (z-scores) | Baseline,  Mean (SD) | Post-intervention,  Mean (SD) |
| --- | --- | --- |
| Global cognition | -0.49 (0.62) | -0.35 (0.66) |
| Executive function | -0.41 (0.53) | -0.34 (0.51) |
| Processing speed | 0.43 (0.56) | 0.38 (0.68) |
| Immediate memory | -1.10 (1.19) | -0.66 (1.31) |
| Delayed memory | -0.63 (1.09) | -0.54 (1.26) |
